# Neural Networks for Semantic and Syntactic Prediction and Visual-Motor Statistical Learning in Adult Readers With and Without Dyslexia

**DOI:** 10.1162/nol.a.8

**Published:** 2025-07-22

**Authors:** Elisa Gavard, Valérie Chanoine, Franziska Geringswald, Jean-Luc Anton, Eddy Cavalli, Johannes C. Ziegler

**Affiliations:** CNRS, CRPN, Aix-Marseille Université, Marseille, France; CNRS, ILCB, Aix-Marseille Université, Aix-en-Provence, France; CNRS, LPL, Aix-Marseille Université, Aix-en-Provence, France; CNRS, Centre IRM-INT@CERIMED, Aix-Marseille Université, Marseille, France; EMC, Université Lumière Lyon 2, Lyon, France

**Keywords:** dyslexia, prediction, reading, semantics, sequence learning, syntax

## Abstract

Prediction has become a key concept for understanding language comprehension, language production, and more recently reading. Recent studies suggest that predictive mechanisms in reading may be related to domain-general statistical learning (SL) abilities that support the extraction of regularities from sequential input. Both mechanisms have been discussed in relation to developmental dyslexia. Some suggest that SL is impaired in dyslexia with negative effects on the ability to make linguistic predictions. Others suggest that dyslexic readers rely to a greater extent on semantic and syntactic predictions to compensate for lower-level deficits. Here, we followed these two research questions in a single study. We therefore assessed the effects of semantic and syntactic prediction in reading and SL abilities in a population of university students with dyslexia and a group of typical readers using fMRI. The SL task was a serial reaction time (SRT) task that was performed inside and outside the scanner. The predictive reading task was performed in the scanner and used predictive versus nonpredictive semantic and syntactic contexts. Our results revealed distinct neural networks underlying semantic and syntactic predictions in reading, group differences in predictive processing in the left precentral gyrus and right anterior insula, and an association between predictive reading and SL, particularly in dyslexic readers. These findings contribute to our understanding of the interplay between SL, predictive processing, and compensation in dyslexia, providing new insights into the neural mechanisms that support reading.

## INTRODUCTION

Prediction is a fundamental cognitive mechanism that allows the brain to anticipate incoming information based on previous experience and contextual cues, facilitating efficient processing in various domains, including language and reading (Favier et al., 2021; Ferreira & Chantavarin, 2018). In reading, this mechanism underpins the rapid integration of linguistic information, allowing readers to seamlessly process text despite the inherent challenges posed by the linear nature of linguistic input (Chang et al., 2006; Pickering & Garrod, 2013; Staub, 2015). Psycholinguistic research has traditionally focused on lexical predictability, showing that word predictability derived from contextual cues modulates word-level processing (Kutas & Federmeier, 2011). However, lexical predictability is fairly rare in connected text, that is, no more than 5% of content words can be predicted from prior context (Luke & Christianson, 2016). However, even if people can rarely predict the precise upcoming word, they tend to be quite accurate in predicting its semantic category or its grammatical role in the sentence, which we will refer to as semantic and syntactic prediction (Carter et al., 2019; Gavard & Ziegler, 2022; Luke & Christianson, 2016).

A major research question is whether making linguistic predictions is related to our capacity to make nonlinguistic predictions, in particular, our ability to extract and exploit statistical regularities in sequential input, also referred to as statistical learning (SL; Misyak & Christiansen, 2012; Saffran et al., 1996). It has been shown that SL is a key mechanism underlying language acquisition (Saffran et al., 1996) and its role in reading development and skilled reading is increasingly discussed (Frost et al., 2015). A particularly strong hypothesis is that impaired SL might be directly related to language learning disorders, including developmental dyslexia (Seidenberg & MacDonald, 2018).

In the present study, we address two research questions that may initially seem contradictory. First, we examine whether the ability to make efficient linguistic predictions and their neural underpinnings are associated with domain-general SL abilities in a visual-motor sequence learning task. Specifically, we investigate whether adults with developmental dyslexia exhibit deficits in SL (Gabay et al., 2015), which could negatively impact their ability to make semantic and syntactic predictions. Second, we explore whether university students with dyslexia rely more heavily on semantic and syntactic predictions to compensate for lower-level orthographic processing deficits (Cavalli, Colé, et al., 2017; Corkett & Parrila, 2008; Stanovich, 1980). These research questions generate different predictions that have previously been examined in separate studies. The present study integrates them within a single framework, using the same tasks and participants.

### Semantic and Syntactic Prediction in Reading

When encountering the sentence “Since it was raining, I took my …” readers seem to predict the upcoming word “umbrella” quite naturally. This form of prediction is called *lexical prediction*. A large number of studies using eye movements, event-related brain potentials and various behavioral paradigms (cloze probability, priming, etc.) have investigated this effect showing word predictability effects, facilitatory priming, reduced N400s to expected words and so on (Federmeier & Kutas, 1999; Kutas & Federmeier, 2011). However, as mentioned above, in connected text, lexical predictions occur much less frequently than one might think as less than 5% of content words can be predicted from prior context (Luke & Christianson, 2016). However, readers can reliably predict both the semantic category or attributes of an upcoming word within a sentence context (e.g., an object to protect you from the rain because “it was raining”) and the syntactic structure (i.e., a noun after “I took my”). That is, semantic and syntactic information is highly predictable even when word identity is not, and this information facilitates reading over and above the predictability of the full word form, as shown by eye movements during reading (see also Bonhage et al., 2015; Henderson et al., 2016). Carter et al. (2019) suggested that “the prediction of semantic information is an automatic, graded process that occurs normally during reading as contextual information builds up over time” (p. 230), while syntactic predictability reflects a reader’s implicit sensitivity to grammatical constraints, learned through repeated exposure to the structure of a language.

Neuroimaging studies have revealed distinct but overlapping networks supporting semantic and syntactic predictions, with semantic prediction associated with regions such as the left inferior frontal gyrus (IFG), middle temporal gyrus (MTG) and angular gyrus, and syntactic prediction involving areas, such as the left anterior temporal lobe and posterior superior temporal sulcus (Carter et al., 2019; Friederici et al., 2003; Pallier et al., 2011). Thus, there is evidence that semantic and syntactic predictions rely on partially different brain networks, but not many studies have tried to disentangle their contributions while controlling for lexical predictions (for an exception, see Dapretto & Bookheimer, 1999).

### Statistical Learning in Reading

A growing body of research suggests that language learning and language processing are supported by domain-general SL skills that enable individuals to extract and exploit statistical regularities from their environment (Misyak & Christiansen, 2012; Saffran et al., 1996). In reading, SL has been discussed in the context of learning the mapping between orthography, phonology, and semantics (Seidenberg & MacDonald, 2018). To our knowledge, the link between SL and the ability to make efficient linguistic predictions has rarely been studied (Gavard & Ziegler, 2024). Ren et al. (2023) conducted a meta-analysis that revealed a moderate relationship between SL and language-related measures, highlighting its developmental variability and modality independence. Indeed, the association between SL and reading or language-related skills was not different for linguistic or nonlinguistic SL paradigms, supporting the idea that the link between SL and language is domain-general (Frost et al., 2019).

A large number of SL tasks have been used, ranging from the classic segmentation tasks (Saffran et al., 1996) to artificial grammar learning (Reber, 1967) and implicit visual-motor sequence learning (for reviews, see Frost et al., 2019; Siegelman & Frost, 2015). Visual-motor sequence learning, as measured in the so-called Serial Reaction Time (SRT) task (Nissen & Bullemer, 1987), is interesting with respect to reading because reading contains visual-motor sequence learning and skill automatization. In the SRT task, participants must respond to stimuli that appear sequentially in different locations on a screen, typically by pressing a corresponding key as quickly and accurately as possible. Over time, participants implicitly learn the repeating sequence of stimuli, which facilitates faster and more accurate responses, demonstrating the acquisition of sequence-specific knowledge. Indeed, previous research has found correlations between performance in the SRT task and reading ability (Arciuli & Simpson, 2012; Gabay et al., 2015; Gavard & Ziegler, 2024).

Neuroimaging studies further showed an overlap between brain regions involved in SL and those involved in reading (Christiansen et al., 2012; Hung et al., 2018, 2019). For instance, Hung et al. (2019) found that regions such as the occipitotemporal cortex, superior temporal gyrus, IFG, and putamen were active both in the SRT and natural reading tasks, with individual differences in reading skills associated with activations in the right insula and right IFG. In addition, eye-tracking measures in the SRT task (i.e., anticipatory saccades) showed that participants used learned sequences to predict upcoming stimuli, with saccadic amplitude shown to be sensitive to sequence learning effects (Lum, 2020). These findings suggest that once visual-motor sequences are learned (i.e., SL), they are used to predict upcoming events (i.e., prediction). However, despite the growing evidence linking SL to reading and language processing, the precise nature of this relationship remains unclear (Frost et al., 2019). It is also unclear whether there is a direct link between SL and the ability to make semantic or syntactic predictions during reading.

### Dyslexia and Compensation

Developmental dyslexia is characterized by persistent difficulties in decoding and word recognition, often attributed to phonological processing deficits that result in inefficient orthographic processing (Snowling & Melby-Lervåg, 2016; Ziegler et al., 2009). Recent frameworks increasingly emphasize a multideficit model, which posits that dyslexia arises from the interaction of multiple cognitive, linguistic, and environmental risk factors (McGrath et al., 2020; Pennington, 2006; Perry et al., 2019; Ziegler et al., 2020). Some dyslexic readers (DRs), particularly those who pursued university studies, have demonstrated the ability to attain normal text comprehension skills, even while continuing to face challenges with lexical and orthographic processing. Thus, we define compensated DRs as those who attained normal text comprehension skills and close-to-normal reading fluency despite persistent deficits in orthographic and phonological processing (i.e., impaired phoneme awareness, rapid automatized naming, decoding, phonological short-term memory). It has been argued that they do so by using compensatory strategies (Cavalli, Duncan, et al., 2017; Stanovich, 1980). Two major compensatory mechanisms have been put forward. First, it has been argued that compensated DRs rely to a greater extent on sentence context and the meaning of a sentence to counter deficits in orthographic processing (Stanovich & West, 1981, 1983). Such a compensation strategy implied that top-down information is used to facilitate bottom-up processing, which is a key feature of human information processing and reading (Grossberg & Stone, 1986). The hypothesis can be reframed in the context of the more recent predictive coding framework to suggest that compensated DRs might use semantic and syntactic predictions to a greater extent than typical readers (TRs) to compensate for low-level deficits. Indeed, behavioral studies have shown that DRs benefit more from sentence-level context than TRs (Corkett & Parrila, 2008; Stanovich, 1980). At the individual word level, DRs have been found to rely more heavily on morphological and morpho-semantic cues to aid comprehension (Cavalli, Duncan, et al., 2017; Elbro & Arnbak, 1996; Frith & Snowling, 1983).

The second mechanism is referred to as neural compensation. The idea is that in order to compensate for the inefficient left-lateralized occipitotemporal reading network, the brain uses additional neural networks (e.g., right hemisphere, frontal regions). This could result in the activation of brain areas outside the classic reading network (Martin et al., 2015). Indeed, adult DRs tend to show increased activation in frontal regions and the left angular gyrus, possibly reflecting an increased reliance on meaning-based cues during reading (Cavalli, Colé, et al., 2017; Price & Devlin, 2011). Other studies have reported increased activation in right occipitotemporal regions, suggesting alternative neural pathways that support reading (Cavalli, Chanoine, et al., 2024). Neural compensation can also be seen in a spatio-temporal reorganisation of neural networks and temporal dynamics with earlier morpho-semantic activations in frontal regions in adult DRs as compared to TRs (Cavalli, Colé, et al., 2017).

### Statistical Learning in Dyslexia

Given the importance of SL in language learning and its potential role in reading (see above), it is obvious that SL became a key candidate for a domain-general deficit that could possibly explain developmental dyslexia (Schmalz et al., 2017; Seidenberg & MacDonald, 2018). Indeed, some studies reported deficits in SL tasks across auditory and visual modalities (Gabay et al., 2015; Ozernov-Palchik et al., 2023) or binocular motor behavior (Przekoracka-Krawczyk et al., 2017). For example, auditory SL tasks, such as syllable-sequence learning, reveal impairments in both children and adults with dyslexia (Gabay et al., 2015). Similarly, Chinese children with dyslexia performed significantly worse than their age-matched controls on both visual SL and orthographic awareness tasks and both of these tasks correlated with reading ability (Tong et al., 2019). As concerns the SRT task, a meta-analysis of 14 studies found that individuals with dyslexia have worse sequence learning abilities (effect size = 0.45) than TRs (Lum et al., 2013). Together, these findings suggest that SL deficits in developmental dyslexia seem to be modality-independent, although variations in task design, age and comorbidity with other neurodevelopmental disorders might moderate the observed effects (van Daal & van der Leij, 1999).

Neuroimaging studies that investigated the link between SL tasks and developmental dyslexia have shown weaker activation in brain areas involved in sequence learning in DRs compared to TRs (Menghini et al., 2006; Nicolson et al., 1999) and suggested that hippocampal function may be associated with sequential procedural learning deficits in developmental dyslexia (Krishnan et al., 2016). However, other studies have not identified a clear link between dyslexia and SL, particularly when examining auditory or nonsequential forms, such as artificial grammars (Inácio et al., 2018; Staels & Van den Broeck, 2017). This lack of association has also been observed in sequential procedural learning tasks involving motor sequences (Henderson & Warmington, 2017). These findings suggest that while DRs may initially struggle with SL, they can achieve similar proficiency levels under supportive conditions, highlighting the importance of task design in evaluating SL abilities. In their critical review of the literature on SL and dyslexia, Schmalz et al. (2017) concluded “that there is insufficient high-quality data to draw conclusions about the presence or absence of an effect” (p. 147) and more recent reviews came to similar conclusions (Schmalz et al., 2017; Singh & Conway, 2021).

### The Present Study

The present study aimed to elucidate the neural mechanisms underlying semantic and syntactic predictions in reading, the potential link between linguistic prediction efficiency and SL, and the use of linguistic prediction as a compensatory strategy in adult DRs. We assessed the efficiency of making linguistic predictions using the recently developed predictive reading task (Gavard & Ziegler, 2022, 2024).

In the predictive reading task, participants are asked to read aloud a final word in a sequence that was preceded by a semantically related context (orange – red – blue – purple – yellow) or a syntactically related context (the – big – carpet – is – yellow). The two conditions were compared to semantically unrelated (tomato – shirt – tulip – cloud – yellow) or syntactically scrambled contexts (the – is – carpet – big – yellow). The semantic context was set up in a way such that participants could easily predict the general semantic category of the final word (e.g., a color) but not the exact lexical item (e.g., yellow). The semantic associations were calculated with word embeddings (Mikolov et al., 2013). The syntactically predictive context was a short syntactically correct sentence. Syntactic predictability was calculated using the Universal Dependencies corpus (de Marneffe et al., 2021). For each target word, we calculated conditional probabilities of predicting the grammatical class of the target word considering the preceding context (P(target/context) > 0.60). Importantly, in the syntactic context, neither the final word nor the semantic category of the final word could be predicted, and this was verified with word embeddings.

To investigate the potential link between linguistic prediction and SL, we used an SRT task (Nissen & Bullemer, 1987), which is a classic SL task that correlates with reading ability (Lum et al., 2013). We actually used a modified version of the SRT task, which allowed us to separate the SL process from the expression of SL (Frensch et al., 1998; Seidler et al., 2005). Specifically, participants were first trained to master a visual-motor sequence, and once the sequence was fully learned, we used fMRI to compare brain responses to the learned (i.e., predictable) sequences with those to scrambled sequences. This paradigm seemed much closer to our predictive reading task, in which we also compared predictable versus scrambled sequences of words, for which the semantic and syntactic associations must have been learnt previously.

The main hypotheses were as follows: First, we hypothesized that semantic and syntactic predictions in reading could be dissociated and would engage distinct neural networks. Second, we expected university students with dyslexia to rely more heavily on semantic and syntactic predictions compared to TRs. Third, we anticipated greater activation in brain regions possibly outside the classic reading network in dyslexic students relative to TRs, suggesting neural compensation. Fourth, if only some DRs were compensated, we predicted these patterns would primarily be observed in DRs with strong reading abilities, leading to interactions between group and reading ability. Fifth, we expected a relationship between SL and reading ability, with potential correlations and overlap in the neural networks associated with both. Finally, we hypothesized that DRs would show poorer sequence learning in the SRT task and weaker neural activation when comparing learned sequences to scrambled sequences in the scanner if developmental dyslexia were linked to SL deficits.

## MATERIALS AND METHODS

### Participants

We recruited 25 typical adult readers (12 males and 13 females) and 25 adults with dyslexia (6 males and 19 females) aged between 19 and 30 years (23.3 ± 3.84 for TRs and 21.4 ± 2.43 for dyslexic). All participants were university students, right-handed and native speakers of French. They were recruited at Aix-Marseille University (France) from a wide variety of academic programs (48% of the DRs and 56% of the TRs were enrolled in humanities and social sciences programs; 52% and 44% were enrolled in natural sciences programs, respectively). They reported no history of psychiatric or neurological disorders, no current use of any psychoactive medications, and normal or corrected-to-normal hearing and vision. Participants gave written informed consent and received €50 for their participation. The experiment was conducted in accordance with the Declaration of Helsinki and received ethical approval (filed under Id 2017-A03614-49 from the regional ethical committee, Comité de Protection des Personnes Sud Méditerranée I).

University students with dyslexia were recruited following a formal diagnosis established either by a regional reference center for learning disabilities (Centre de Référence des Troubles des Apprentissages [Center for the Diagnosis of Learning Disabilities]) at the AP-HM Hospital in Marseille or by the specialized disability support service (Mission Handicap) of Aix-Marseille University’s medical department. All participants had received a diagnosis of dyslexia during primary school and undergone remedial teaching. Additionally, they reported significant difficulties in learning to read during childhood and adolescence, which were confirmed before inclusion in the study using the French version of the Adult Reading History Questionnaire—Revised (ARHQ-R; Lefly & Pennington, 2000). This standardized self-report questionnaire is widely used to screen for dyslexia in adults. To be included in the study, all participants with dyslexia had to score above the cutoff threshold of 0.47 (Cavalli, Brèthes, et al., 2024). The ARHQ-R consists of 23 Likert-scale items addressing various aspects of literacy and educational experiences, such as reading habits, reading, and spelling abilities, reading speed, attitudes toward school and reading, additional academic support received, grade repetition, and the effort required to succeed in elementary, secondary, and post-secondary education, as well as in current life.

All participants completed a set of neuropsychological tasks (see Table 1 for group comparisons), including assessment of nonverbal IQ (using the Raven Progressive Matrices; Raven, 2000) and verbal IQ (using the Vocabulary subtest of the Wechsler Adult Intelligence Scale—Fourth Edition; Wechsler, 2008). Participants with a formal diagnosis of specific language impairment or other conditions potentially impacting language abilities (e.g., autism spectrum disorder) were excluded from the present study. The neuropsychological assessment also included reading and reading-related tasks assessing skills known to be persistently impaired in adults with dyslexia, including those who successfully pursue university studies (Brèthes et al., 2022). These tasks were recently developed and standardized for adults with and without dyslexia (see Cavalli, Brèthes, et al., 2024, for validity and reliability measures) and included: a 1-minute word reading task, a 2-minute pseudoword reading task, a connected-text reading fluency task, and a phonemic awareness task. Additionally, we administered the Alouette unconnected-text reading fluency test, which is considered a “gold standard” in France for diagnosing dyslexia in children, adolescents, and adults (see Cavalli et al., 2018). All materials and detailed descriptions of the tasks are available on the Open Science Framework website (https://osf.io/zmf82/).

**Table 1.** Characteristics of the typical readers (TR) and the dyslexic readers (DR)

	Group				*F*
	TR (*n* = 25)		DR (*n* = 25)		
	*M*	*SD*	*M*	*SD*	
Chronological age	23.32	3.84	21.36	2.43	4.66*
ARHQ-R	0.29	0.11	0.56	0.08	100.74***
Reading					
Connected-text reading fluency^a^	221.00	40.32	157.32	33.71	36.70***
Unconnected-text reading fluency^b^	533.18	79.02	379.66	89.41	41.38***
Single word reading^b^	126.77	22.94	89.00	16.82	22.08***
Pseudoword reading^b^	162.23	38.24	89.52	24.19	64.55***
Phonemic awareness^b^	2.07	0.39	1.35	0.55	28.07***
Intelligence					
Verbal IQ	10.84	3.14	9.48	3.28	2.24
Nonverbal IQ	47.96	6.02	42.48	6.24	10.00**

*Note*. ARHQ-R = Adult Reading History Questionnaire—Revised (Lefly & Pennington, 2000).

^a^ Number of correctly read words in 1 min.

^b^ Efficiency score based on accuracy (A) and reading time in seconds (RT): (A/RT) * maximum RT.

* *p* < 0.05.

** *p* < 0.01.

*** *p* < 0.001.

The participants’ characteristics and group comparisons on the neuropsychological tasks are summarized in Table 1. On the neuropsychological assessment, which included word, pseudoword and text reading tasks, the ARHQ, and the phonemic awareness task, adult DRs displayed significant impairments. Specifically, 23 out of 25 TRs (91%) scored above the cutoffs, and 22 out of 25 DRs (80%) scored below the cutoffs. While the two groups did not differ significantly in verbal IQ, a significant difference was observed in nonverbal IQ, with DRs displaying lower scores compared to TRs. However, it is important to note that all participants scored above the fifth percentile on both nonverbal and verbal IQ. This ensures that none of the participants exhibited deficits in nonverbal reasoning or oral language.

### Stimuli and Procedure

Each participant performed a single fMRI session including a T1-weighted anatomical scan, five functional runs in the predictive reading task, three functional runs in the SRT task, and one functional run in the localizer. Stimuli were projected onto a semitranslucent projection screen at the rear of the scanner by a PROPixx DLP LED projector (VPixx Technologies, Saint-Bruno, QC, Canada) with a resolution of 1,920 × 1,080 pixels and a frame rate of 60 Hz and could be viewed through a mirror mounted on the MRI head coil just above the eyes. The visible area of the image subtended 500 × 440 mm, covering 20 × 18° of visual angle. Participants’ eye position was recorded using an Eyelink 1000 Plus Long-Range Mount eye tracker (SR Research Ltd, Mississauga, Ontario, Canada), using corneal reflection and pupil tracking, with a temporal resolution of 1000 Hz. At the beginning of the fMRI session, the eye tracker was calibrated using a 5-point gaze-calibration. Before each functional run, the spatial accuracy of the eye tracker was validated using the same five points and the calibration was redone if the average deviation exceeded 1° of visual angle. Participants’ responses for the predictive reading task were recorded using OptoAcoustics’ FOMRI-III, an MRI-compatible optical audio recording system with real-time amplification and noise cancellation. Participants’ responses for the SRT task were recorded using an ergonomic five-button keypads (right hand) with precise measurement of reaction time (RT) using electronic counters. Stimulus presentation, response registration and synchronization were implemented using LabVIEW (National Instrument). Participants were allowed self-determined breaks after each functional run.

#### Predictive reading task

**Figure 1.**
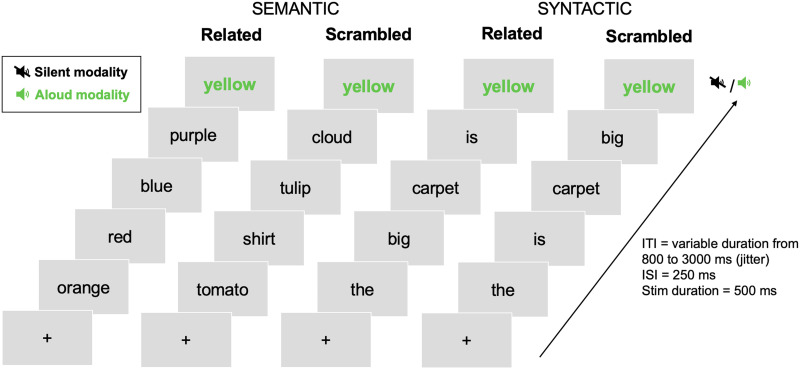
Illustration of the experimental procedure and stimuli in the predictive reading task that was performed in the scanner. *Abbreviations*: ITI = intertrial interval; ISI = interstimulus interval; Stim = stimulation.

The design of the predictive reading task is illustrated in Figure 1. In the semantic condition, participants had to read a final target word (highlighted in green) that was preceded by a context of semantically related or unrelated nouns (orange – red – blue – purple – yellow vs. tomato – shirt – tulip – cloud – yellow). The semantic relatedness between all context words was calculated using a distributional vector space model of semantic associations trained on a very large number of words (Frwiki, 11GB, 914,601,321 tokens, DISCO; Kolb, 2008). In the syntactic condition, the final word of the sequence was preceded by a context of syntactically correct or incorrect word sequences (the – big – carpet – is – yellow vs. the – is – carpet – big – yellow). The syntactic contexts were constructed such that the target word was strongly predicted on a syntactic basis but not at all on a semantic basis (verified using the same vector space model as above). Syntactic predictability was calculated using the Universal Dependencies corpus (de Marneffe et al., 2021). For each target word, we calculated conditional probabilities of predicting the grammatical class of the target word considering the preceding context (P(target/context) > 0.60). Eighty syntactically nonpredictive contexts were created by using the same target words but scrambling the words of the context such that syntactic prediction was close to zero (P(target/context) < 0.01). We used 160 French target words with frequencies ranging from 1 to 158 per million (*M* = 13.63, *SD* = 26.4; New et al., 2004), and lengths ranging from 3 to 10 letters (*M* = 6.44, *SD* = 1.7). The design, materials, and calculation of semantic relatedness and syntactic prediction strength were the same as reported in Gavard and Ziegler (2022).

A scanning session consisted of five functional runs. A run comprised 64 trials, including eight repetitions of each condition (semantically related versus unrelated, syntactically related versus unrelated, reading task silent versus aloud). The toolbox NeuroDesign (2017) was used to optimize the intertrial interval (ITI) and the order of the trials with respect to contrast estimation and minimization of the variance of the design matrix (Kao et al., 2009; Wager & Nichols, 2003). We created five different schedules for stimulus presentation, one for each functional run, that were constrained to a maximum of three consecutive repetitions of each condition and used ITIs drawn from an exponential distribution (minimum 0.8, mean 1, and maximum 3 s). The schedules were counterbalanced across participants. In total, participants read 320 trials distributed evenly across conditions and runs. The words were presented in 60-point Arial font, in black or green against a light grey background. Each trial started with a fixed cross presented at the center of the screen for a variable duration from 800 to 3,000 ms (jitter), followed by a sequence of five words. Each word was displayed for 500 ms with a 250 ms interstimuli interval. Participants were instructed to read silently words in black and read aloud words indicated in green as quickly as possible. For half of the trials, the target word was indicated in green, for the other half the target word was displayed in black, which indicated no reading aloud response (see Figure 1). The dependent variable was the RT for the words colored in green, that is, the time between the appearance of the green word and the participant’s response.

#### Serial reaction time task

**Figure 2.**
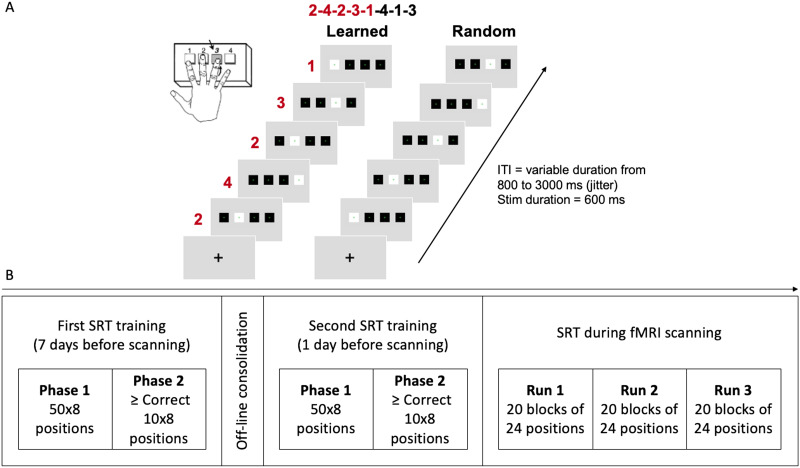
Serial Reaction Time (SRT) task. (A) Experimental design and timing of the SRT task in the fMRI scanning adapted from Schendan et al. (2003). In the training session, participants had to press the S, R, T, and J keyboard buttons with the same four fingers of the right hand as in the subsequent fMRI experiment. (B) Experimental procedure of the SRT task in the training and scanner sessions. *Abbreviations*: ITI = intertrial interval; ISI = interstimulus interval; Stim = stimulation.

To investigate SL abilities, we used a modified version of the SRT task (Nissen & Bullemer, 1987). The SRT task is a visual-motor sequence learning task, in which participants are presented with visual stimuli at different locations on a screen and have to press response keys corresponding to the location of the stimulus (see Figure 2A). Unbeknownst to the participants, the order of stimulus locations follows a fixed sequential pattern during a training phase. Implicit learning of this sequence leads to speeded responses compared to control blocks, during which stimulus locations are presented randomly.

##### Training session.

Participants were trained to execute an 8-digit finger sequence with their right hand over a period of 1 week before the fMRI scanning. A first training session took place 7 days before the fMRI scanning and a second session 1 day before the fMRI scanning (see Figure 2B). A fixed second-order conditional sequence was used, ideal for studying implicit sequence learning (Schwarb & Schumacher, 2012). Participants had to perform the sequences as fast and accurately as possible. The training session consisted of a first phase during which 50 repetitions of the sequences were presented. To ensure an equal level of learning, the initial phase was followed by a second adaptive phase, which was stopped after the participants had performed 10 sequences in a row without making any mistakes (see Figure 2B). Participants were instructed to press the keyboard buttons S, R, T, J as fast and accurately as possible with the four fingers of the right hand (from index to little finger). The finger positions corresponded to the positions of the target (white square) on the screen. The two trainings were performed at home using LabVanced, a unified JavaScript framework for online studies (Finger et al., 2017). The two dependent variables were accuracy and RT for correctly performed sequences.

##### During fMRI scanning.

Participants performed the same eight-digit finger sequences learned in the training sessions at home (learned condition) with their right hand on an MRI-compatible keyboard, with keys numbered 1–4 for the four fingers of the right hand excluding the thumb. Blocks of the learned condition were intermixed with blocks of the random condition. In the random condition, the positions were presented the same number of times at each of the four locations as in the learned condition and were matched with respect to the frequency of the first-order transitions (1-3, 1-4, 2-3, 2-4, 3-1, 3-2, 4-1, 4-2). This ensured that the direction as well as the distance that the visual stimulus moved between successive locations was the same for the learned and the random conditions which was crucial for the analysis of the eye movement parameters. Importantly, these constraints also ensured that potential differences between the learned and random conditions were due to learning complex patterns of successive positions, such as second-order transitions or higher (segments of three or more positions) and could not be due to differences in lower-level properties of the sequences (frequencies of single locations or first-order transitions). A scanning session consisted of three functional runs. Within each run, 10 blocks containing three repetitions of the eight positions of the learned sequence (learned condition) and 10 blocks containing 24 positions of the pseudo-random sequences (random condition) were presented. Each block started with a black fixation cross presented at the center of the screen for a variable duration from 800 to 3,000 ms (jitter), followed by 24 successive images indicating the target responses. The images were composed of three black squares and one white square representing the target position on a light-grey background and were each displayed for 600 ms. The start positions of each block were chosen pseudo-randomly and counterbalanced across the learned and random condition. We used NeuroDesign toolbox to create three different schedules for stimulus presentation optimizing the ITI (exponential distribution; minimum 0.8, mean 1, and maximum 3 s) and the order of blocks that were constrained to a maximum of three repetitions of the same condition and counterbalanced across participants. As in the training, participants were instructed to press the key on the keyboard corresponding to the position of appearance of the target (white square) on the screen as fast and accurately as possible (see Figure 2). Participants were not informed of the repetitions prior to training and scanning. The two dependent variables were accuracy and RT for correctly performed sequences.

#### Localizer task (in scanner)

The localizer task was adapted from a 5-min fast acquisition procedure designed by Pinel et al. (2007), which captures the cerebral bases of auditory and visual perception, motor actions, reading, language comprehension and mental calculation at an individual level. Eight types of stimuli were used: flashing horizontal and vertical checkboards, visual and auditory motor instructions (e.g., “Press three times the left button”), visual and auditory sentences (e.g., “We easily find a taxi in Paris”), visual and auditory subtractions (e.g., “Subtract nine to eleven”). Each type of stimuli was presented in 10 successive trials in a randomized order for each participant. Only the contrast between reading sentences versus viewing flashing checkerboards was retained for the subsequent analyses. This was done to define a subject-specific functional region of interest (ROI) representing the reading network.

### Data Acquisition

The experiment was conducted on a Siemens Magneton Prisma 3T scanner at the Centre IRM-INT@CERIMED (UMR 7289, CNRS, Aix-Marseille University) using a 64-channel head coil. The anatomical images were acquired using high-resolution structural T1-weighted image (repetition time [TR] = 2.3 s, echo time [TE] = 2.98 ms, voxel size = 1 mm × 1 mm × 1 mm, flip angle = 9°, field of view [FOV] = 256 × 240 × 192 mm^3^, matrix size = 256 × 240 × 192). A fieldmap acquisition (TR = 7.305 s, TE = 59 ms, voxel size = 2 mm × 2 mm × 2 mm, flip angle = 90°, FOV = 200 × 200 × 144 mm^3^, matrix size = 100 × 100 × 72) was collected to estimate and correct the B0 inhomogeneity. The functional images were acquired using a T2*-weighted gradient-echo planar sequence with 72 slices per volume (TR = 1.39 s, TE = 33.4 ms, multiband acceleration factor = 4, voxel size = 2 mm × 2 mm × 2 mm, flip angle = 56°, FOV = 200 × 200 × 140 mm^3^, matrix size = 100 × 100 × 72). A total of 1,025 functional scans were acquired over five runs (4.8 min per run) for the reading task, 711 functional scans were acquired in three runs (5.3 min per run) for the SRT tasks, and 233 functional scans were acquired in one run (5.22 min) for the localizer task.

### Data Preprocessing

We preprocessed the fMRI data using fMRIPrep (Version 21.0.2; Esteban et al., 2019), a robust and standardized pipeline, which applies distortion correction, realignment, and normalization to MNI (Montreal Neurological Institute) space. A detailed preprocessing report can be created automatically (see https://fmriprep.org/en/21.0.2/workflows.html) and is included in the Supplementary Material, available at https://doi.org/10.1162/nol.a.8.

### Data Analysis

#### Behavioral and eye movement data analysis

As age and nonverbal IQ were not perfectly matched between our groups, we included them as covariates in the analyses of the behavioral data.

##### Predictive reading task.

The reading aloud responses were recorded in the scanner. Therefore, the wave files had to be denoised to determine the onset time. We then used CheckVocal (Protopapas, 2007) to determine the onset of each reading aloud response (RT) and judged whether the word was pronounced correctly. As explained in Whole-Brain Analysis below, one dyslexic participant (sub-49) was entirely excluded from the analyses. Out of all 7,840 observations (RTs), 186 were removed because of missing data (2.37% of the data). We then excluded RTs exceeding 1,500 ms (0.55% of the data), and we further considered as outliers data points that were above or below 2.5 *SD*s from each individual participant’s mean RT. Outliers were replaced by the cutoff RT corresponding to each participant’s ±2.5. *SD* (Hoaglin et al., 1986). For the RT analysis, we used R (Version 4.2.2; R Core Team, 2022) and lme4 (Bates et al., 2015) to perform linear mixed-effects models (LMMs). We report unstandardized regression coefficients (b), standard errors (*SE*s), *p* values, and standardized beta coefficients.

##### Serial reaction time task.

We used R (Version 4.2.2) to perform two LMM analyses, first with accuracy as the dependent variable, and then with RT of correct responses as the dependent variable. Out of all 70,560 observations (RTs), 14,188 were removed because of incorrect responses (20.20% of the data). A significant proportion of the incorrect responses (5,796 out of 14,188) were due to response mismatches, probably caused by time discrepancies. Excluding these misaligned responses reduced the rate of truly incorrect responses to 11.9% of the total data. However, to ensure methodological rigour, all incorrect responses, including misaligned and truly incorrect responses, were excluded from the analyses to avoid potential confounding and to ensure an accurate assessment of participants’ performance. We report unstandardized regression coefficients (b), *SE*s, *p* values, and standardized beta coefficients.

##### Eye movements.

In addition to the manual response button presses, we analysed saccadic responses to the visual stimuli as a more direct mapping between the visual cues and the anticipated response in the learned sequences in the SRT (Karatekin et al., 2007; Lum, 2020; Marcus et al., 2006). We excluded blocks in which more than 25% of the eye movement data were missing due to signal loss or eye blinks and runs in which more than 50% of the blocks were missing. Following this procedure, as explained in Region of Interest Analysis, the data from one dyslexic participant (sub-49) was entirely excluded (100% missing data for all runs) and three runs from two controls and four runs from three dyslexic participants were not considered for analysis (60%–90% missing data per run). For the remaining data, on average 19.5 blocks for controls (mean signal loss 2.30%) and 18.7 blocks for dyslexic participants (mean signal loss 4.47%) were entered in the analysis. Saccades were identified from the eye-tracking data with the EyeLink Data Viewer (SR Research, 2019), using velocity and acceleration thresholds of 35°/s and 9500°/s^2^, respectively. We included only saccades that had a minimum duration of 10 ms, whose start and end points fell within the screen area and that were directed to the left or to the right. To extract a score of predictive saccade performance, we defined a temporal window ranging from −300 to 200 ms around the target square onset. The distance of the landing point of the first saccade that was initiated within this temporal window was then analysed with respect to the center of the white square in the visual stimulus indicating the target position. Predictive saccades were expected to result in smaller distances of the landing points to the target square specifically in the learned sequences compared to the random condition. Overall individual scores of predictive saccades as an indicator of SL performance were calculated for each participant as the difference between the mean distance of the saccade landing position to the target square in the blocks of the learned condition and the mean distance between the saccade landing position to the target square of the random condition in the blocks and averaged across runs. Lme4 was used to perform LMM and GLMM analyses and lmerTest was used to perform *t* tests using Satterthwaite approximation.

##### Correlation between tasks.

Because we were interested in the relation between SL, linguistic prediction and reading ability, we conducted a series of correlation analyses using R and the Hmisc (Harrell, 2025) and PerformanceAnalytics (Peterson, 2019) packages. Individual prediction scores in the predictive reading task were calculated by dividing for all participants the difference between their speed on scrambled minus related trials by their overall response speed. Individual SRT learning scores in the SRT task (training session at home) were calculated by dividing for all participants the difference between their speed on first training (7 days before scanning) minus second training (1 day before scanning) of the second phase (participant dependent learning) by their overall response speed. Individual SRT scores in the SRT task (during fMRI scanning) were calculated by dividing for all participants the difference between their speed on random minus learned sequences by their overall response speed. Individual SRT saccades scores in the SRT task (during fMRI scanning) were calculated by dividing for all participants the difference between their mean distance saccade (degrees of visual angle [deg VA]) on random minus learned sequences by their overall mean distance. For reading ability, we selected the Alouette reading test (a measure of reading fluency) as it exhibited the strongest factor loadings in a principal component analysis conducted across all administered neuropsychological tasks.

#### Whole-brain analysis

The postprocessing was performed with Statistical Parametric Mapping software (SPM12; Ashburner et al., 2021) on MATLAB R2022b (MathWorks, 2022). The pre-processed images were spatially smoothed with a 4 mm full width at half-maximum isotropic Gaussian kernel and normalized in the MNI space MNI152NLin2009cAsym. The brain masks included grey matter, white matter, and cerebral spinal fluid. Prior to the first level analysis, the functional data in the first level model were high-pass filtered with a cutoff of 128 s. Stimulus-specific blood oxygen level dependent (BOLD) effects were estimated by convolving the stimulation with the canonical hemodynamic response function.

For the first level, we used a general linear model (GLM) in SPM12 for each task with 12 nuisance regressors related to a head motion estimation (6 linear and 6 quadratic functions related to translation and rotation movements). Additionally, 10 physiological regressors were also used as nuisance regressors from fmriprep to allow for component-based noise correction (CompCor; Behzadi et al., 2007): five anatomical principal components from WM and five from CSF. Then, we added one more nuisance regressor related to spike (outliers) from time series data. The number of spikes was calculated based on two cutoffs of the time series data (find_spike function from nltools library from Python): one global spike cutoff identifying spikes in the global signal in standard deviations and one difference spike cutoff identifying spikes in the average frame difference in standard deviations. For excessive motion, we have adapted the procedure described in Siegel et al. (2014), censoring individual frames with framewise displacement > 0.5 and standardized version of DVARS (std_DVARS) > 2 (Power et al., 2012). If more than 15% of the frames in a run were censored, then the entire run was omitted (Etzel, 2023). Finally, one dyslexic participant (sub-49) was entirely excluded from analysis based on the participant’s difficulty in remaining motionless during the acquisition, resulting in the omission of more than two runs calculated with the procedure used above (the only run of the localizer task and two runs out of three of the SL task). We estimated the GLM with the following regressors of interest for each of the three tasks. (1) Predictive reading task: it included eight regressors of interest modeling the condition (semantic [sem] vs. syntactic [synt]), the context (related [rel] vs. scrambled [scr]), and the modality (silent [sil] vs. aloud [alo]): [sem_rel_alo], [sem_rel_sil], [sem_scr_alo], [sem_scr_sil], [synt_rel_alo], [synt_rel_sil], [synt_scr_alo], [synt_scr_sil]. (2) SRT task: it included two regressors of interest modeling the condition (learned vs. random): [lea], [ran]. (3) Localizer task: it included 10 regressors of interest modeling the type of stimuli used: [audio_computation], [audio_left_hand_button], [audio_right_hand_button], [horizontal_checkerboard], [vertical_checkerboard], [sentence_listening], [sentence_reading], [visual_computation], [visual_left_hand_button], [visual_right_hand_button].

Statistical parametric maps for each experimental factor and each of the 49 participants were calculated at the first level and then entered a second-level one-sample *t* test analysis of variance (random effects analysis using a threshold at the voxel-level of 0.001 without correction for multiple correction). Whole-brain analysis results are displayed after controlling for the family-wise error (FWE) at 0.05 for multiple comparisons at cluster-level.

#### Region of interest analysis

The postprocessing was similar to the univariate whole-brain analysis; however, the images were not spatially normalized or smoothed to take advantage of the high spatial frequency pattern information within each participant’s data (Kriegeskorte et al., 2006). All ROIs were converted into the native space of each participant using the inverse of the transformation matrix that was used to normalize the T1 image into the standard MNI152NLin2009cAsym space (using the ANTS library in python).

##### Anatomical ROIs.

**Figure 3.**
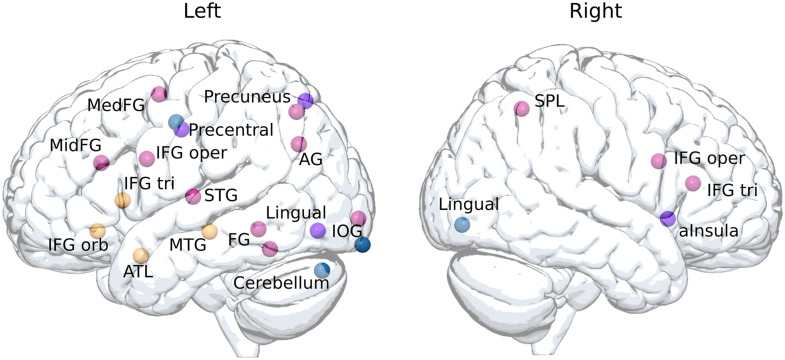
Illustration of the regions of interest (ROIs). For the predictive reading network, anatomical ROIs were extracted from two recent articles on linguistic predictions (Carter et al., 2019; Schuster et al., 2020). The regions related to word predictability are shown in yellow, those related to semantic predictability in purple, and those related to syntactic predictability in blue. For the single word reading network, anatomical ROIs were extracted from a recent meta-analysis (Murphy et al., 2019) and are shown in pink. ROI spheres are projected respectively on left and right sides of MNI cortical surface. *Abbreviations*: AG = angular gyrus; aInsula = anterior insula; ATL = anterior temporal lobe; FG = fusiform gyrus; IFG oper = inferior frontal gyrus pars opercularis; IFG orb = IFG pars orbitalis; IFG tri = IFG pars triangularis; IOG = inferior occipital gyrus; MedFG = medial frontal gyrus; MidFG = middle frontal gyrus; MTG = middle temporal gyrus; SPL = superior parietal lobule; STG = superior temporal gyrus.

ROIs based on MNI coordinates were identified from recent studies on word predictability (Schuster et al., 2020), semantic/syntactic predictability (Carter et al., 2019), and from a meta-analysis on single word reading (Murphy et al., 2019). They are shown in Figure 3. For the regions assumed to be related to word predictability, the following four ROIs located on the left hemisphere were defined: the IFG pars triangularis (IFG tri; *x* = −48, *y* = 18, *z* = 6), the IFG pars orbitalis (IFG orb; *x* = −45, *y* = 30, *z* = −9), the anterior temporal lobe (*x* = −48, *y* = 9, *z* = −21), and the MTG (*x* = −54, *y* = −24, *z* = −9). For the regions assumed to be related to semantic predictability, the following four ROIs were considered: the left precentral gyrus (*x* = −49.5, *y* = −10.5, *z* = 40.5), the left precuneus (*x* = −1.5, *y* = −73.5, *z* = 52.5), the left lingual gyrus (*x* = −1.5, *y* = −79.5, *z* = −10.5), and the right anterior insula (*x* = 34.5, *y* = 16.5, *z* = −4.5). For the regions assumed to be related to syntactic predictability, the following four ROIs were selected: the left precentral gyrus (*x* = −52.5, *y* = −13.5, *z* = 37.5), the left occipital cortex (*x* = −25.5, *y* = −100.5, *z* = −16.5), the left cerebellum (*x* = −40.5, *y* = −79.5, *z* = −28.5), and the right lingual gyrus (*x* = 1.5, *y* = −85.5, *z* = −7.5). For the regions assumed to be related to single word reading, the following 14 ROIs were considered: the left middle frontal gyrus (MidFG, *x* = −40, *y* = 28, *z* = 24), the left medial frontal gyrus (MedFG, *x* = −4, *y* = −2, *z* = 56), the bilateral IFG pars opercularis (IFG oper; *x* = −49, *y* = 11, *z* = 18; *x* = 50, *y* = 14, *z* = 20), the bilateral IFG tri (*x* = −48, *y* = 18, *z* = 6; *x* = 50, *y* = 29, *z* = 13), the left precentral gyrus (*x* = −50, *y* = −8, *z* = 44), the left precuneus (*x* = −22, *y* = −68, *z* = 48), the left angular gyrus (*x* = −44, *y* = −68, *z* = 33), the left superior temporal gyrus (STG; *x* = −54, *y* = −16, *z* = 8), the left fusiform gyrus (FG1; *x* = −40, *y* = −48, *z* = −8; FG2; *x* = −42, *y* = −54, *z* = −18), the left inferior occipital gyrus (IOG; *x* = −24, *y* = −98, *z* = −4), and the right superior parietal lobule (*x* = 34, *y* = −56, *z* = 50). ROIs with an MNI coordinates center and a 6-mm radius were first created as a binary mask using the MarsBaR SPM toolbox (Brett et al., 2002). They were then converted into the native space (see above) and resampled to fit the spatial resolution of functional images previously defined in task-based GLM.

##### Functional ROIs.

Functional ROIs were defined from statistical and individual t-maps using a voxel-level threshold of *p* < 0.001 without correction for multiple comparisons and with an extent threshold of 5 voxels, to generate binary masks. These masks were then transformed into native space. One functional ROI was employed in this study: deltaLoc corresponding to the contrast Sentence Reading − Checkerboards in the localizer task.

The parameter estimates (beta values) were extracted from each ROI using MarsBaR toolbox and were used to calculate the percent signal change (PSC). PSC values were computed from the trial signal against the global signal (see https://marsbar-toolbox.github.io/faq.html#how-can-i-extract-the-percent-of-activated-voxels-from-an-roi) and averaged (by the median) across voxels within an ROI.

From each experimental condition, we thus obtained one value of PSC per participant and per ROI. In the predictive reading task, we considered two levels of prediction: PSem for Semantic-Related Prediction − Semantic-Scrambled Prediction, and PSyn for Syntactic-Related Prediction − Syntactic-Scrambled Prediction. In the localizer task, one contrast was retained: Sentence Reading − Checkerboards.

For the PSC analysis, we used R (Version 4.2.2) and lme4 (Bates et al., 2015) to perform an LMM analysis with participants as random effects and (1) prediction, group, and reading ability (behavioral score of the Alouette standardized, see Participants) as fixed effects for the predictive task; and (2) group and a behavioral measure of the SRT task (Saccade, SRT score, and SRT learning) for the link between the predictive reading and the SRT tasks. *Saccade* is a behavioral score of the oculomotor measure (see Whole-Brain Analysis), *SRT score* is a behavioral score of the SL ability during fMRI scanning (difference between RTs on Random − Learned sequences), and *SRT learning* is a behavioral score of the sequence learning ability in the training session at home (difference between RTs on First − Second Training session). We report unstandardized regression coefficients (b), *SE*s, *p* values, and standardized beta coefficients.

## RESULTS

### Behavioral Results

The inclusion of age and nonverbal IQ as covariates did not change any of the main results, and no significant effects of the covariates were observed. We, therefore, removed these covariates from the final models to reduce model complexity.

#### Predictive reading task

**Figure 4.**
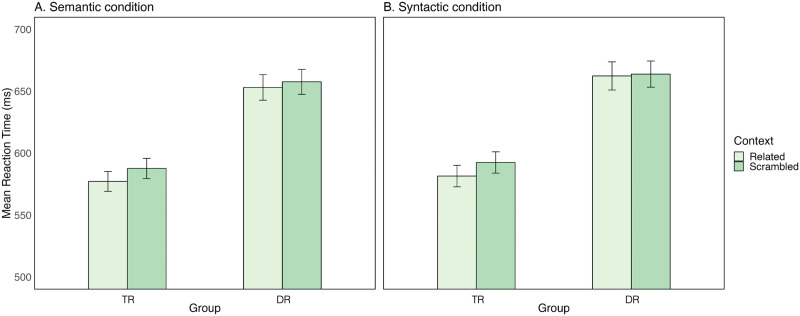
Performance in the predictive reading task. Mean reaction times (ms) as a function of group (typical readers [TR] vs. dyslexic readers [DR]) and context (related vs. scrambled) for the (A) semantic and the (B) syntactic condition. Error bars indicate 95% confidence intervals.

Participants did not make any naming errors. Thus, accuracy was not further analysed. Because the RT distribution was slightly skewed, we log-transformed the RTs. This resulted in a normal RT distribution (Skewness: 0.29, Kurtosis: −0.09; Blanca et al., 2013). For the LMM, the model included the maximum random structure that allowed convergence (Barr, 2013). The final model included condition, context, group, and all interactions between these variables. As random effects, we included by-participants intercepts and random slopes for condition and context by participants (RT ∼ condition * context * group + (1 + condition * context | subject). The results showed significant effects of context (*b* = 0.008, *SE* = 0.003, *p* < 0.05, *β* = 0.07) and group (*b* = 0.051, *SE* = 0.020, *p* < 0.05, *β* = 0.41), but no effect of condition nor an interaction between the fixed effects. Planned contrast analysis showed that TRs, unlike the DRs, were faster at reading the target word following related than unrelated contexts in both the semantic (*b* = −0.008, *SE* = 0.003, false discovery rate [FDR]-adjusted *p* < 0.05) and syntactic (*b* = −0.009, *SE* = 0.004, FDR-adjusted *p* < 0.05) conditions (see Figure 4). No significant context effects were found for DRs, neither in the semantic, nor the syntactic condition (all *p* > 0.05).

#### Serial reaction time task

##### Training session.

While DRs were slower (*b* = −62.44, *SE* = 31.4, *p* < 0.05) and made more errors (*b* = 5.28, *SE* = 1.69, *p* < 0.01) when learning the sequences during the first phase of the training session (during which 50 repetitions of the sequences were presented), they became as fast (*b* = −0.05, *SE* = 0.03, *p* = 0.07) and accurate (*b* = −376, *SE* = 211, *p* = 0.08) as the TRs during the second phase of the training session (which was stopped after the participants had performed 10 sequences in a row without making any mistakes). Overall, DRs needed more time to reach the same level of performance during home training, ∼4 min versus ∼3 min, for DRs versus TRs, respectively (*b* = −22,237, *SE* = 9,960, *p* < 0.05). The LMM for the RTs of the second phase of the training session included session (first training session vs. second training session), group, and the interactions between these variables. The models included the maximum random structure that allowed convergence (Barr, 2013). As random effects, we included by-participants intercepts and random slopes for session by participants (RT ∼ session * group + (1 + session | subject). The results showed a significant effect of session (*b* = −0.09, *SE* = 0.01, *p* < 0.001, *β* = 0.65), that is, both TRs and DRs were faster in the second session (1 day before scanning), but no effect of group and no interaction between the fixed effects, showing that an equal level of learning was achieved for all participants.

##### During fMRI scanning.

**Figure 5.**
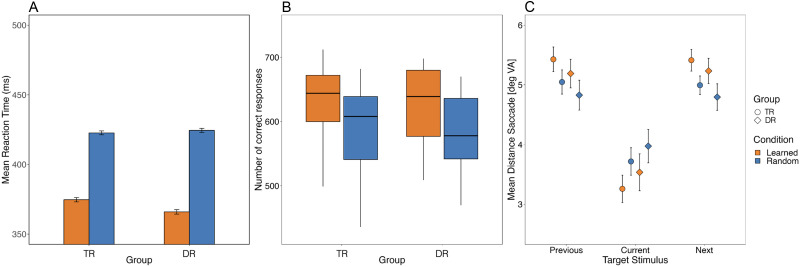
Behavioral results of the SRT task in the scanner. (A) Mean reaction time (ms) as a function of group (TRs vs. DRs) and condition (learned vs. random). (B) Accuracy (raw score on 720 correct responses) as a function of group and condition. (C) Mean distance of saccade landing position to stimulus center in degrees of visual angle (deg VA) as a function of target stimulus (previous, current, next), separated for TRs (circles) and DRs (diamonds) and learned (orange) and random condition (blue). Errors bars indicate 95% confidence intervals.

Because the RT distribution was slightly skewed, we log-transformed the RTs. This resulted in a normal RT distribution (Skewness: −0.56, Kurtosis: 0.13). For the LMM, the models included the maximum random structure that allowed convergence (Barr, 2013). The final models included condition, group, and the interactions between these variables. As random effects, we included by-participants intercepts and random slopes for condition by participants. The LMM for the RTs (RT ∼ condition * group + (1 + condition | subject) showed a significant effect of condition (*b* = 0.06, *SE* = 0.007, *p* < 0.001, *β* = 0.46) but no effect of group nor an interaction between the fixed effects. Contrast analysis showed that both TRs and DRs were faster in the learned than in the random condition (TR: *b* = −0.06, *SE* = 0.007, FDR-adjusted *p* < 0.05; DR: *b* = −0.07, *SE* = 0.008, FDR-adjusted *p* < 0.05) (see Figure 5A). The LMM for the accuracy (Perf ∼ condition * group + (1 + condition | subject) showed a significant effect of condition (*b* = −17.16, *SE* = 2.65, *p* < 0.001, *β* = 0.54) but no effect of group nor an interaction between the fixed effects. Contrast analysis showed that both TRs and DRs were more accurate in the learned than in the random condition (TR: *b* = 17.2, *SE* = 2.65, FDR-adjusted *p* < 0.001; DR: *b* = 20.7, *SE* = 2.70, FDR-adjusted *p* < 0.001; see Figure 5B).

##### Eye movements.

We first performed a GLMM on the number of saccades per block to test whether saccade number differed between condition or group. The GLMM included condition, group, and the interaction as fixed effects and by-participants intercepts and random slopes for condition by participants as random effects. This analysis did not reveal any significant effects (all absolute *b* < 0.025, *p* > 0.35), indicating that the overall number of saccades was comparable (control, random 26.4 saccades; control, learned 26.1 saccades; dyslexic, random 26.7 saccades; dyslexic, learned 25.9 saccades). To investigate whether saccades were predictive in the learned sequences, we analysed the distance of the post-saccadic fixations, that is, the Euclidean distance of the saccade landing position to the center of the target square for the first saccade that was initiated during a time window ranging from 300 ms prior to visual stimulus onset to 200 ms post-stimulus onset. Note that due to the temporal constraints of the fMRI design, it was not possible to include blanks during the SRT task. The previous target stimulus was thus still present on screen during the initial 300 ms of the temporal window. We performed three LMM analyses comparing the distance of the saccade landing positions with respect to the previous stimulus, the current stimulus, and the next stimulus of the sequences. If saccades were predictive in the learned sequences, we expected significantly reduced distances to the current stimulus, and significantly increased distances to the previous and next stimulus specifically for the learned condition. The models included condition, group, and the interaction as fixed effects and by-participants intercepts and random slopes for condition by participants as random effects (deg VA ∼ condition * group + (1 + condition | subject). For all three analyses, condition was the only significant effect (distance to previous stimulus, *b* = 0.381, *SE* = 0.063, *p* < 0.001; distance to current stimulus, *b* = −0.444, *SE* = 0.066, *p* < 0.001; distance to next stimulus, *b* = 0.416, *SE* = 0.058, *p* < 0.001) and followed the expected pattern. On average, saccade end positions were closer to the target center in the learned condition by 0.46 and 0.44 degrees of visual angle for TRs and DRs respectively, but they were further away from the center of the previous stimulus by 0.38 and 0.36 degrees and by 0.42 and 0.44 degrees for the next stimulus for TRs and DRs respectively (see Figure 5C).

#### The link between predictive reading and SL

The correlations between the predictive reading task and the SRT task are presented in Table 2 for the DRs (above the diagonal) and the whole sample (below the diagonal). Note that we found no significant correlations for the TRs only. The analyses showed no significant correlation between semantic or syntactic prediction (or the average of the two) and the SRT measures. However, we found a significant positive correlation between the reading ability of the DRs and the overall predictive reading measure (*r* = 0.49, *p* < 0.05, FDR corrected) and with the syntactic predictive measure (*r* = 0.49, *p* < 0.05, FDR corrected). This positive correlation means that DRs who scored higher on the standardized reading tests were also better linguistic predictors, specifically in the syntactic condition. We also found a significant positive correlation between the reading ability of the DRs and the performance (accuracy) in the SRT task during fMRI scanning (*r* = 0.54, *p* < 0.05, FDR corrected). Thus, DRs who scored higher on the standardized reading tests were also better statistical learners. Note that these correlations could also be interpreted the other way around: good linguistic predictors or good statistical learners tend to be better readers. Finally, we found a significant positive correlation between the SRT score and the anticipatory saccades in the SRT task (*r* = 0.55, *p* < 0.05, FDR corrected), which means that DRs who were better predictors in the SRT task were also better at anticipating the positions of targets at the oculomotor level.

**Table 2.**
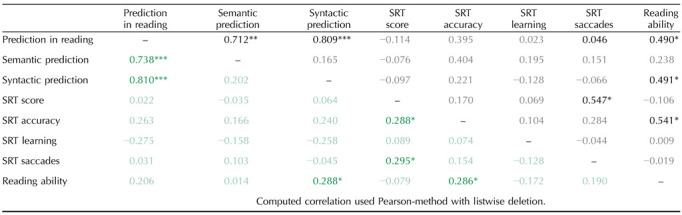
Correlation coefficients between the SRT variables and the predictive reading variables

*Note*. Correlation coefficients for dyslexic readers are presented in black (above the diagonal) and for the whole sample in green (below the diagonal). Whole sample in green. Significant correlations are marked by asterisks: *p < 0.05, **p < 0.01, ***p < 0.001 (FDR corrected).

### Whole-Brain Results

We performed one-sample *t* tests to obtain the mean activation of each contrast for the three tasks (predictive reading task, SRT task, and localizer) in each group (TRs and DRs). We compared the activation of each contrast between the two groups with two-sample *t* tests. Statistically significant effects at the whole-brain level were identified using a voxel-level threshold of *p* < 0.001 (uncorrected) and a cluster-level threshold of *p* < 0.05 (FWE corrected for multiple comparisons). Significant activations per voxel or per cluster were labeled using the Neuromorphometrics atlas in SPM12.

#### Localizer task

**Figure 6.**
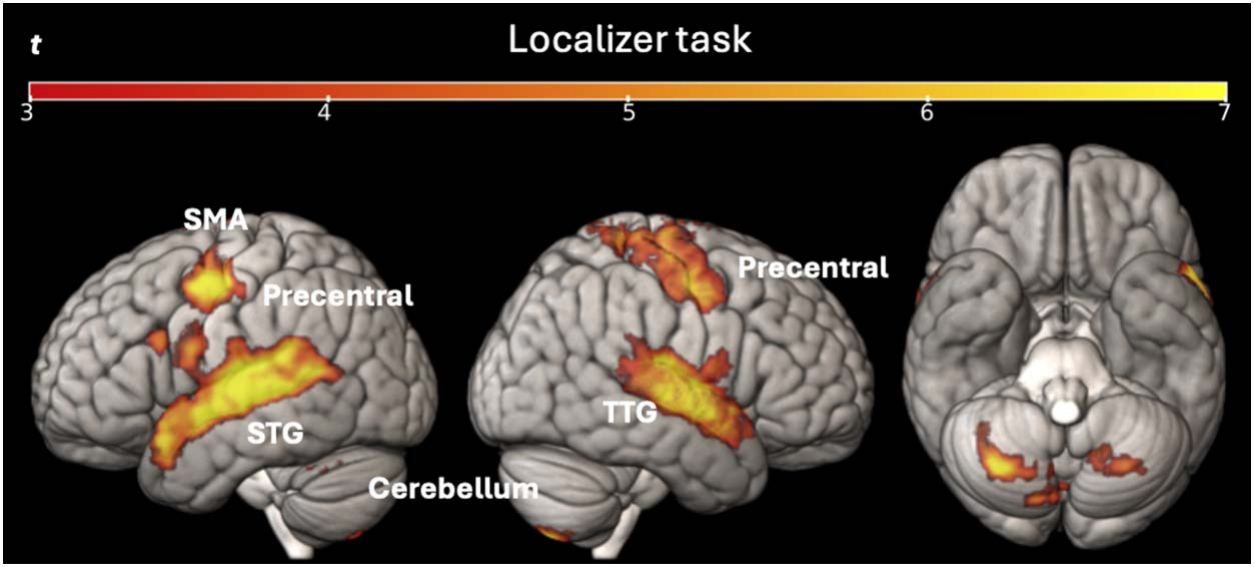
Univariate whole-brain results of sentence reading in the localizer task (Sentence Reading − Checkerboards) for all participants. Statistical t-maps are projected respectively on left, right, and below sides of cortical surfaces (from MNI standard human cortex) using an uncorrected voxel-wise threshold of *p* < 0.001 and a cluster-wise threshold with FWE correction of *p* < 0.05. *Abbreviations*: SMA = supplementary motor cortex; STG = superior temporal gyrus; TTG = transverse temporal gyrus.

The contrast Sentence reading − Checkerboard revealed a similar activation profile for TRs and DRs and was in line with the results reported by Pinel et al. (2007). The two-sample *t* test did not show any significant differences between the two groups in silent sentence reading (at a voxel-level statistical threshold of *p* < 0.001 without correction, and a cluster-level threshold of *p* < 0.05 with FWE correction). We found a significant activation mainly in the left STG and the right transverse temporal gyrus, in the bilateral precentral gyrus, the left supplementary motor cortex, the bilateral precuneus, the left caudate, and the bilateral cerebellum exterior (see Figure 6). The full list of activation clusters (brain areas, extent, *t* values and coordinates) is available in Supplementary Table S1.

#### Predictive reading task

For the main effect of group (TRs vs. DRs), we conducted two-sample *t* tests for all the contrasts resulting from the eight regressors of interest described in the Data Analysis section. The two-sample *t* test did not show any significant differences between the two groups for any of the contrasts (at a voxel-level statistical threshold of *p* < 0.001 without correction, and a cluster-level threshold of *p* < 0.05 with FWE correction).

**Figure 7.**
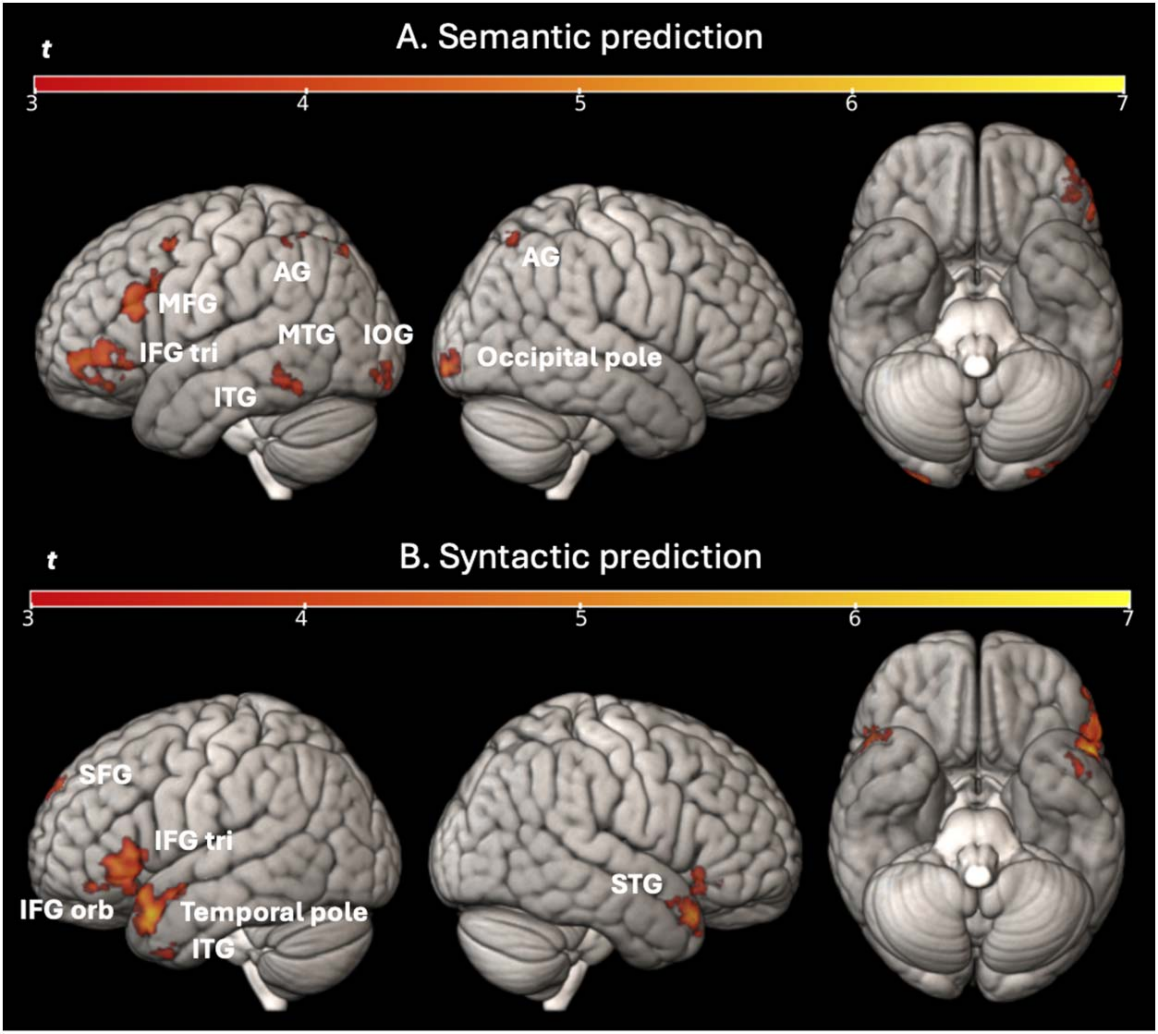
Univariate whole-brain results in the predictive reading task for (A) semantic and the (B) syntactic prediction (related minus scrambled) for all participants. Statistical t-maps are projected respectively on left, right, and below sides of cortical surfaces (from MNI standard human cortex) using an uncorrected voxel-wise threshold of *p* < 0.001 and a cluster-wise threshold with FWE correction of *p* < 0.05. *Abbreviations*: AG = angular gyrus; IFG orb = IFG pars orbitalis; IFG tri = IFG pars triangularis; ITG = inferior temporal gyrus; MFG = middle frontal gyrus; SFG = superior frontal gyrus; STG = superior temporal gyrus.

For the main effect of prediction (see Figure 7), we found a significant activation for semantic prediction (Semantically Related − Unrelated) mainly in the middle frontal gyrus, the left IFG tri (BA45), the left MTG, the bilateral inferior temporal gyrus, the left supramarginal gyrus, the bilateral angular gyrus, the left caudate, the left IOG and the right occipital pole. For the syntactic prediction (Syntactically Related − Scrambled), we found a significant activation mainly in the left superior frontal gyrus, the left IFG tri (BA45) and IFG pars orbitalis (IFG orb; BA47), the left temporal pole, the right MTG (right temporal pole), the right cuneus, the right lingual gyrus, the left posterior insula and the left putamen. The full list of activation clusters (brain areas, extent, *t* values and coordinates) is available in Supplementary Table S2 and S3.

#### Serial reaction time task

**Figure 8.**
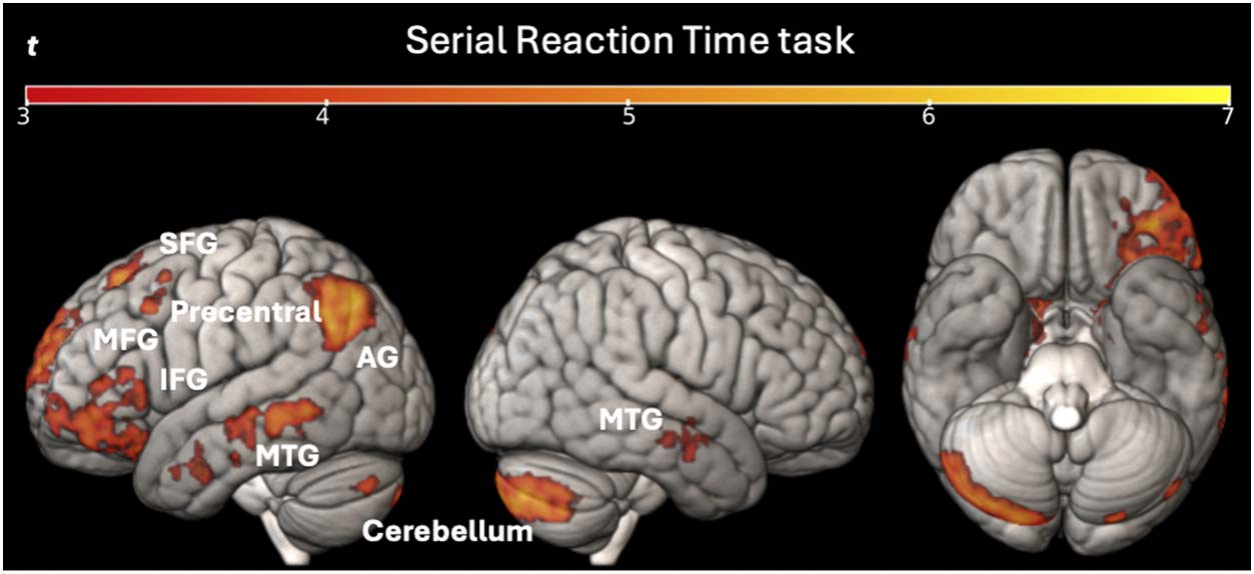
Univariate whole-brain results for Learned − Random sequences in the SRT task for all participants. Statistical t-maps are projected respectively on left, right, and below sides of cortical surfaces (from MNI standard human cortex) using an uncorrected voxel-wise threshold of *p* < 0.001 and a cluster-wise threshold with FWE correction of *p* < 0.05. *Abbreviations*: AG = angular gyrus; IFG = inferior frontal gyrus; MFG = middle frontal gyrus; MTG = middle temporal gyrus; SFG = superior frontal gyrus.

As in the predictive reading task, the two-sample *t* test did not show any significant differences between the two groups for any of the contrasts resulting from the two regressors of interest described above in the Data Analysis section (at a voxel-level statistical threshold of *p* < 0.001 without correction, and a cluster-level threshold of *p* < 0.05 with FWE correction). The one-sample *t* test on condition (Learned − Random) showed activation mainly in left superior frontal gyrus, the left middle frontal gyrus, the left IFG, the left precentral gyrus, the left bilateral, the left angular gyrus, the right lingual gyrus, the bilateral MTG, the right cuneus, the bilateral thalamus, the left amygdala, the bilateral caudate, the right putamen, and the bilateral cerebellum exterior (see Figure 8). The full list of activation clusters (brain areas, extent, *t* values and coordinates) is available in Supplementary Table S6.

### Region of Interest Results

#### Predictive reading task

**Figure 9.**
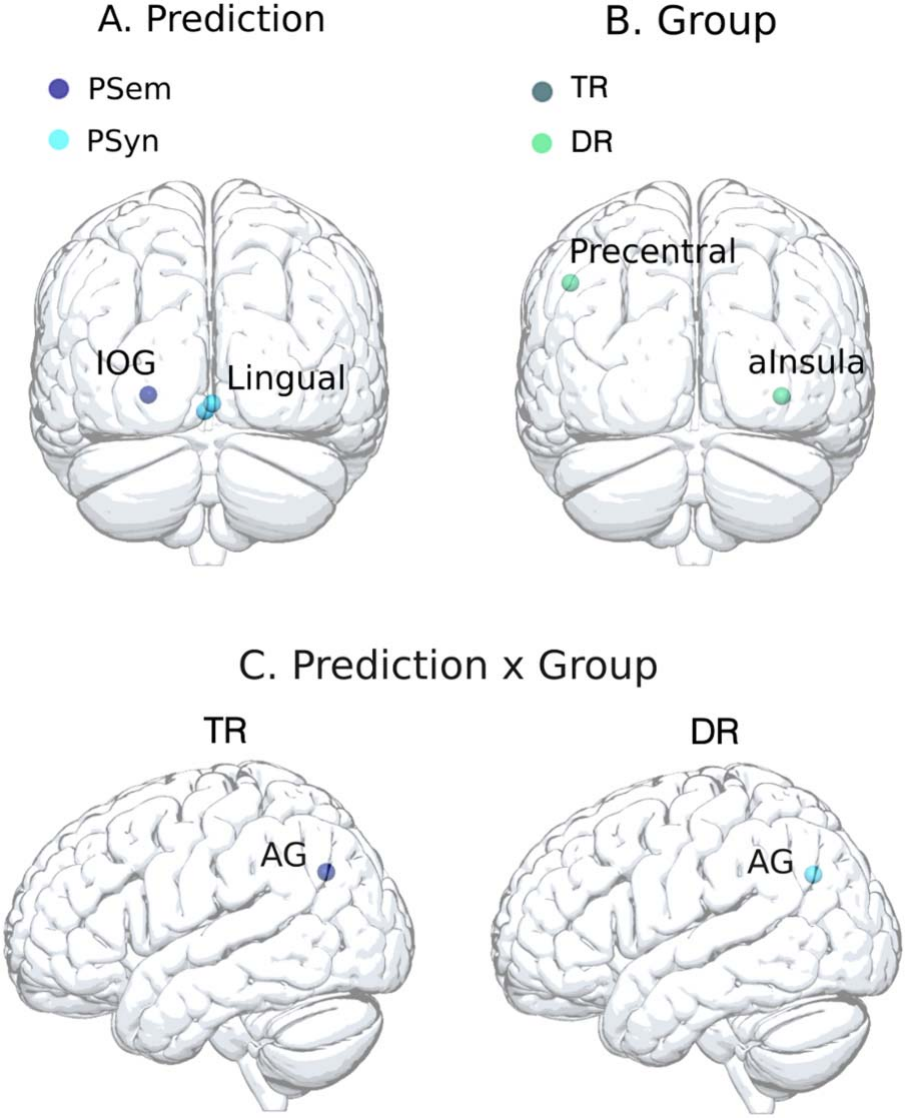
Effects of semantic and syntactic predictions (PSem vs. PSyn) and their interactions for typical (TR) and dyslexic readers (DR). Only significant effects per ROI are illustrated. ROIs with significant (A) prediction effects (semantic compared to syntactic in dark blue and syntactic compared to semantic in light blue) and (B) group effects (TRs compared to DRs in dark green and DRs compared to TRs in light green) are projected on the posterior view of the brain. ROIs with significant (C) interaction between the effects of prediction and group are projected on the left view of the brain. *Abbreviations*: AG = angular gyrus; aInsula = anterior insula; IOG = inferior occipital gyrus.

In the ROI analyses, we used PSC in the various ROIs at the dependent variable. The effects were analysed using LMMs. The parameter estimates (beta values) were extracted from each ROI using MarsBaR toolbox and were used to calculate the PSC. The model included the maximum random structure that allowed convergence (Barr, 2013). The final model included prediction, group, reading ability, and all interactions between these variables. As random effects, we included by-participants intercepts and random slopes for prediction by participants (PSC ∼ prediction * group * reading ability + (1 + prediction | subject). The results are summarized in Figure 9.

Results showed a significant effect of prediction in the left lingual (*b* = −0.02, *SE* = 0.008, *p* < 0.05, *β* = 0.23), the right lingual (*b* = −0.02, *SE* = 0.008, *p* < 0.05, *β* = 0.24), and the left IOG (*b* = 0.03, *SE* = 0.005, *p* < 0.001, *β* = 0.45); a significant effect of group in the left precentral gyrus (*b* = −0.01, *SE* = 0.006, *p* < 0.05, *β* = 0.20) and in the right anterior insula (*b* = 0.02, *SE* = 0.008, *p* < 0.01, *β* = 0.24); and a significant interaction between the effects of prediction and group in the left angular gyrus (*b* = −0.02, *SE* = 0.006, *p* < 0.05, *β* = 0.19). For TRs, the left angular gyrus was more activated for semantic compared to syntactic predictions, while, for DRs, it was more activated for syntactic predictions. The effect of reading ability and the interaction between the effects of reading ability, prediction, and group were not significant.

#### The link between predictive reading and SL

**Figure 10.**
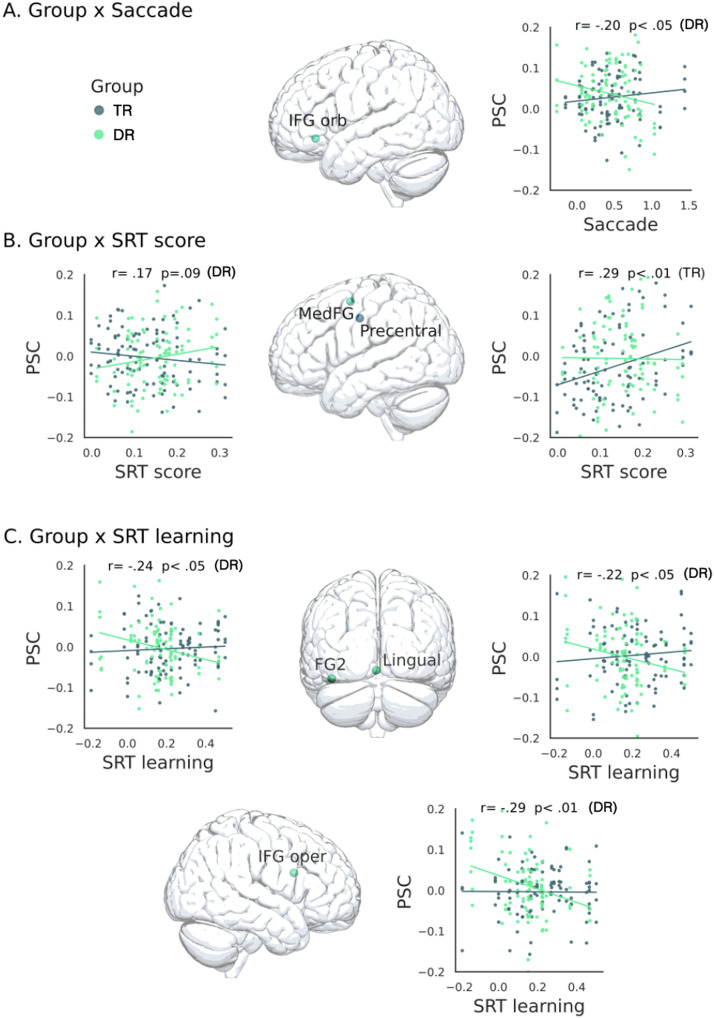
Percent signal change (PSC) in specific ROIs in the predictive reading task as predicted by the behavioral SRT measures (Saccade, SRT score, and SRT learning) and their interaction with group. For the two regression lines of each scatterplot, only the significant correlation coefficients are provided. The scatter plots show (A) ROIs that exhibit a significant interaction between group and saccades, (B) interaction between group and SRT score projected on the left view of human brain, and (C) ROI with significant interactions between group and SRT learning projected on the posterior and right views of human brain. TRs compared to DRs are represented in dark green; DRs compared to TRs are represented in light green. *Abbreviations*: FG = fusiform gyrus; IFG oper = inferior frontal gyrus pars opercularis; IFG orb = IFG pars orbitalis; MedFG = medial frontal gyrus.

We performed three LMM analyses with PSC of the predictive reading task as a dependent variable and the different SRT scores as fixed effects to correlate the BOLD signal change in the predictive reading task with the SRT behavioral measures (SRT score, SRT learning, saccade). As random effects, we included by-participants intercepts and random slopes. The results are summarized in the Figure 10.

The first LMM analysis (PSC ∼ group * saccade + (1 | subject) showed a significant interaction between *group* and *saccade* in the left IFG orb (BA47; *b* = −0.06, *SE* = 0.03, *p* < 0.05, *β* = 0.17). Correlation analysis showed higher PSC in the predictive reading task for DRs who are better at anticipating targets in the SRT task, as revealed by the saccade measure (*r* = −0.20, *p* = 0.05, FDR corrected).

The second LMM analysis (PSC ∼ group * SRT score + (1 | subject) showed a significant interaction between group and SRT score in the left medFG (*b* = 0.28, *SE* = 0.13, *p* < 0.05, *β* = 0.16) and in the left precentral gyrus (*b* = 0.18, *SE* = 0.09, *p* < 0.05, *β* = 0.14). Correlation analyses showed higher PSC in the predictive reading task for DRs with higher SRT scores in the medFG (*r* = 0.17, *p* = 0.09, FDR corrected) and for the TRs with higher SRT scores in the left precentral (*r* = 0.29, *p* < 0.01, FDR corrected).

The third LMM analysis (PSC ∼ group * SRT learning + (1 | subject) showed a significant interaction between group and SRT learning in the right IFG oper (BA44; *b* = 0.08, *SE* = 0.04, *p* < 0.05, *β* = 0.18), the left lingual gyrus (*b* = 0.08, *SE* = 0.03, *p* < 0.05, *β* = 0.17), and the left fusiform gyrus (FG2; *b* = 0.07, *SE* = 0.03, *p* < 0.05, *β* = 0.18). Correlation analyses showed higher PSC for DRs who showed weaker improvement with home practice (right BA44, *r* = −0.29, *p* < 0.01, FDR corrected; left lingual gyrus, *r* = −0.22, *p* < 0.05, FDR corrected; left fusiform gyrus, *r* = −0.24, *p* < 0.05). Note that a higher SRT learning score indicates greater improvement across sessions, suggesting an initial difficulty in sequence learning that improves with practice. Conversely, a smaller SRT learning difference could indicate efficient SL from the start.

## DISCUSSION

The first goal of the present study was to investigate the neural network underlying predictive reading processes and to understand whether making semantic and syntactic predictions in reading is associated with domain-general SL abilities. The second goal was to test whether university students with dyslexia rely to a greater extent on semantic or syntactic prediction than TRs and whether they show evidence for SL deficits, as suggested by previous studies showing SL deficits in dyslexia (Lukasova et al., 2016; Ozernov-Palchik et al., 2023; Przekoracka-Krawczyk et al., 2017). Our hypotheses were the following. First, we hypothesized that semantic and syntactic predictions can be dissociated and recruit distinct neural networks. Second, we hypothesized that university students with dyslexia would rely to a greater extent on semantic and syntactic predictions than TRs. Third, we hypothesized that we should find greater levels of activation in regions possibly outside the classic reading network in university students with dyslexia as compared to TRs. Fourth, we expected interactions between the effects of interest with group and reading ability because the effects should be driven by compensated DRs who would be better readers than non-compensated DRs. Fifth, we expected to see correlations between SL and reading ability and possibly overlap in the neural networks associated with SL and reading. Finally, we expected to find poorer sequence learning in DRs in the SRT task and possibly weaker activation when comparing learnt against scrambled sequences in the scanner. We will now summarize and discuss our findings in the context of the extant literature on reading, dyslexia, and SL.

### Neural Basis of Semantic and Syntactic Prediction in Reading

Regarding our aim to investigate the neural networks associated with semantic and syntactic predictions in reading, the whole-brain analysis showed the following dissociation. For semantic prediction, we found activation in left frontal and temporal areas (left middle frontal gyrus, IFG tri, MTG, and inferior temporal gyrus), but also in the left angular gyrus and the left IOG. For syntactic prediction, we found activation in the left IFG triangularis and orbitalis and in the left temporal areas (as in the studies of Bonhage et al., 2015 and Henderson et al., 2016), in the left MTG (as found by Lopopolo et al., 2017 and Schuster et al., 2016) and in the left superior frontal gyrus. In addition, the ROI analysis showed a greater activation in the semantic compared to the syntactic condition in the left IOG and in the syntactic compared to the semantic condition for the left and right lingual regions. This dissociation is informative for understanding prediction during reading as it suggests that there are two different networks involved in making semantic and syntactic predictions, which is in line with Bonhage et al. (2015), who suggested that linguistic predictions can be based on different sources of high-level linguistic information.

These results are also in line with Dapretto and Bookheimer (1999), who were probably the first to convincingly demonstrate a dissociation between semantic and syntactic neural networks. While previous studies typically manipulated the syntactic complexity of sentences (e.g., Caplan et al., 1998), they managed to create a task, in which the syntactic complexity did not vary across conditions. In their experiment, participants were presented with pairs of sentences and had to judge whether they had the same literal meaning. In the semantic condition, the sentences were identical except that one word was replaced by a synonym. In the syntactic condition, the meaning of the sentence was identical, but the two sentences differed in word order (e.g., active vs. passive voice). They showed that a part of Broca’s area (BA 44) was in charge of computing the syntactic structure, while the lower portion of left IFG (BA 47) was selectively involved in processing the semantic content. This double dissociation provided strong evidence that these linguistic functions are subserved by distinct cortical areas.

In the psycholinguistic literature, both semantic and syntactic prediction effects have been investigated using the priming paradigm, in which a prime influences the reading of a subsequent, related target word (Forster, 1998). While semantic priming effects (“doctor” primes “nurse”) are well established in lexical decision and naming (e.g., Perea & Gotor, 1997), syntactic priming effects (e.g., “the oven” vs. “he oven”) have been studied less and were not always found (Seidenberg et al., 1984). Although it is easy to conceptualize how semantically related words become activated (e.g., automatic spreading of activation), it is more difficult to conceptualize how syntactic information would facilitate the processing of individual words. Yet, there is evidence to suggest that syntactic contexts provide strong constraints on single word identification. For example, Snell and Grainger (2017) reported faster partial report word identification accuracy for words that were embedded in syntactically correct sequences as opposed to words embedded in scrambled agrammatical sequences.

Clearly, the robustness of our context effects in TRs might be due to the fact that we used much longer contexts to build up semantic and syntactic expectations, as opposed to the classic priming studies. At the same time, compared to previous studies, which manipulated syntactic complexity, our task allowed us to dissociate semantic and syntactic processing, much like the study by Dapretto and Bookheimer (1999). The advantage of the present paradigm is that responses were always made to the exact same target word in the different conditions, which eliminates possible confounds when comparing different sentences across conditions. It is also interesting to note that no correlation was found between the scores for semantic prediction and those for syntactic prediction, and these results may suggest that these two types of linguistic prediction are distinct and do not necessarily rely on a common mechanism (Gavard & Ziegler, 2024).

### Is There Evidence for Compensatory Strategies in University Students With Dyslexia?

Based on the previous literature on compensatory strategies in developmental dyslexia (Cavalli, Duncan, et al., 2017; Stanovich, 1980), we made the strong hypothesis that university students with dyslexia would rely to a greater extent on semantic and syntactic predictions than TRs, which should have resulted in larger semantic and syntactic context effects for DRs compared to TRs. However, we found the opposite effect, that is, DRs in our study showed no significant context effects, while TRs showed the classic facilitatory context effect. This finding stands in contrast with previous studies which showed that DRs benefit to a greater extent from sentence-level context than TRs (Corkett & Parrila, 2008; Stanovich, 1980). There are two possible explanations for this discrepancy. First, in our paradigm, context is useful (i.e., predictive) in only half of the trials, the other half being scrambled or unrelated. Given that the context is not always predictive, and that reading is effortful for DRs, they might have decided to ignore the context and focus on the target word, which would result in the absence of context effects. However, this interpretation does not hold up because the fMRI results showed a highly active prediction network in DRs that was not significantly different from that of TRs. What this suggests is that the prediction effects in fMRI might reflect reading comprehension, that is, syntactically correct sentences or semantically related sequences make sense as opposed to scrambled sentences or unrelated sequences. However, the context effects are not strong enough to improve reading aloud of individual words as this bottom-up process is highly deficient in developmental dyslexia. We therefore suggest that we see evidence for neural compensation (i.e., identical predictive networks) for implicit reading comprehension but no behavioral compensation in reading aloud.

Another explanation for the absence of linguistic prediction effects in DRs is that only compensated DRs should show compensatory effects and it may be not all the university students with dyslexia had developed efficient compensatory strategies. The results of the correlation analyses between predictive reading and reading ability are in favour of this interpretation. Indeed, we obtained a significant correlation (*r* = 0.491) for DRs between syntactic prediction and reading ability, suggesting that DRs who are better readers (i.e., more compensated DRs) show stronger syntactic prediction effects (faster RTs for target words in syntactically correct sentences). This correlation provides at least some evidence that more compensated DRs might exploit linguistic context to improve word identification and reading aloud.

Additional support for neural compensation was found through the ROI analysis, which revealed group differences in the left precentral gyrus, left angular gyrus, and right anterior insula. Specifically, greater activation was observed in the left precentral gyrus and right anterior insula in the dyslexic group, while the left angular gyrus showed higher activation for syntactic compared to semantic predictions in the same group. These results suggest that students with dyslexia rely on brain regions associated with high-level linguistic prediction—such as the precentral gyrus for semantic predictions and the angular gyrus for syntactic predictions, as shown in Carter et al. (2019)—likely to enhance sentence-level reading comprehension, rather than focusing on reading aloud.

The present study sheds some new light on the nature of compensation in developmental dyslexia. In DRs, bottom-up information (single word identification) is clearly impoverished (e.g., Ziegler et al., 2014). Our results suggest that linguistic context as a compensatory strategy is not used to improve or “clean-up” the impoverished bottom-up information but rather improve the comprehension of a sentence or a sequence of words despite the presence of impoverished bottom-up information. Compensation is suggested to operate at the sentence level rather than the word level. This idea would explain the recent results by Cavalli, Chanoine, et al. (2024) who investigated whether university students with dyslexia compensate for their reading deficits by a neural reorganization of the typical reading network, where the lexical representations of individual words would be (re-)structured according to semantic rather than orthographic information. They predicted greater similarity between the neural representations of single words (i.e., correlation of neural dissimilarity matrices between individual words) in regions associated with semantic processing and weaker similarity in regions associated with orthographic processing. However, they found the opposite. Adults with dyslexia showed less (rather than more) sensitivity to semantic similarity in the posterior subpart of fusiform gyrus (FG1) in the left hemisphere. However, neural activation was measured for individual words and representational similarity analyses were performed between individual words. If compensation does not affect the neural representation of individual words but rather sentence processing under impoverished input, this would explain why semantic compensation was not seen at the individual word level in the study by Cavalli, Chanoine, et al. (2024).

### Is There a Link Between Linguistic Prediction and SL?

Is the ability to make efficient linguistic predictions linked to the ability to extract statistical regularities from the environment? The adaptive function of SL is often attributed to its role in supporting prediction, where information about an upcoming event is represented prior to its occurrence (Batterink et al., 2025). The whole-brain analysis revealed a large overlap between brain areas activated during the SRT task and the predictive reading task (i.e., the left superior frontal gyrus, the middle frontal gyrus, the IFG, the angular gyrus, the MTG, the right cuneus and the left caudate and the bilateral putamen). In the ROI analysis, we investigated whether the BOLD signal change in the predictive reading task correlated with behavioral measures of SL (SRT saccades, the SRT scores during the fMRI scanning and the SRT learning score during the learning phase before the fMRI scanning). The results highlighted interactions between group and the SL measures with negative correlations for DRs in SRT saccades and SRT learning scores in the bilateral IFG, the left lingual gyrus and in the left fusiform gyrus, and positive correlations in SRT score in the medFG. This pattern suggests an association between better and more accurate performance in anticipating targets in the SRT task and higher predictive reading abilities in DRs.

These findings are consistent with the possibility that individuals who are better statistical learners may also be more effective at using predictive mechanisms during reading. These findings are also consistent with the meta-analysis by Lee et al. (2022), which highlights the critical role of SL in reading development and its moderate association with reading and language-related skills. Importantly, their review emphasizes that SL skills may account for individual differences in reading ability, which is consistent with the patterns of correlations observed in our study. In addition, Tong et al. (2025) provide neuroimaging evidence that SL involves coordinated activity across different brain regions, supporting the idea that SL contributes to prediction by integrating statistical regularities across tasks. Their findings further support the overlap in neural mechanisms between SL and predictive reading observed in our study.

The results also revealed an interaction between group and SRT score, with a positive correlation for TRs in the left precentral gyrus. Interestingly, we found similar results to those of Hung et al. (2019), identifying a network of regions common to both SL and reading, including the fusiform gyrus, precentral area, IFG (pars triangularis and opercularis), and the putamen. This suggests that visual and motor processing, sequence coding (Friederici, 2011), consolidation, and the automatic performance of learned skills (Doyon & Benali, 2005; Nicolson & Fawcett, 2007; Ullman, 2004) may be jointly involved in both visual-motor SL and predictive reading. In addition, in their study, Hung et al. (2019) also found that activation of the right insula and right IFG tri in the reading task and SRT task were associated with individual differences in reading ability. Although we did not find the same relationship with the right insula, we did find greater activation of the right anterior insula in predictive reading in students with dyslexia, suggesting that this structure plays a role in individual differences in (predictive) reading. These findings seem to highlight the potential interaction between general SL mechanisms and reading strategies in dyslexia.

### Is There an SL Deficit in Dyslexia?

We used one of the classic SL tasks, the SRT task, to investigate whether there was an SL deficit in adults with dyslexia. The only evidence for an SL deficit in our SRT task was the finding that it took DRs longer than TRs to learn the visual-motor sequences during the training session. Once both groups had reached the learning criterion (10 consecutive sequences without errors), both groups showed a facilitatory effect of the predictive sequences compared to the random sequences on RTs and accuracy and there were no significant differences between the groups. In addition, in both groups, saccades landed closer to the current target in the learned condition, showing that anticipatory saccades reflect predictive motor planning during SL tasks (Lum, 2020; Lum & Clark, 2022). Importantly, this pattern was observed for both TRs and DRs, suggesting that participants in both groups were able to effectively use their knowledge of learned sequences to anticipate upcoming stimuli.

In the fMRI data, we contrasted the activation pattern of previously learned sequences against random sequences. The whole-brain analysis revealed activation for predictive against random sequences in the left caudate, the left cerebellum, and the right putamen, structures that in the literature appear to play an important role in the early stages of sequence-specific learning in humans (Janacsek et al., 2020). Importantly, there was no group difference in this analysis, suggesting that adult DRs show no deficits in reproducing learned sequences and the neural network they use to do so is not different from TRs.

Where does this leave us? As noted previously, we used a modified version of the SRT paradigm, which allowed us to separate the sequence learning process from the sequence recall process, that is, SL from the expression of SL (Frensch et al., 1998; Seidler et al., 2005). Although there was some evidence for poorer SL—it took the DRs significantly longer than the TRs to reach the performance criterion—we found absolutely no difference between the two groups neither in behavior nor in fMRI with respect to the expression of SL (recall). Given that developmental dyslexia is a learning disorder, poorer performance in the learning process is an important result that is evidence in favour of an SL deficit in the SRT task (Du & Kelly, 2013; Lum & Clark, 2022; Lum et al., 2013). More work is needed to better understand the dissociation between the learning and the expression of SL in developmental dyslexia.

### Conclusion

Our results reveal distinct neural networks for semantic and syntactic predictions during reading, supporting the dissociation between semantic and syntactic processes reported by Dapretto and Bookheimer (1999). We found that university students with dyslexia did not use semantic and syntactic predictions to compensate for lower level orthographic and articulatory processes involved in reading aloud single words. However, their brains processed predictive sentences and sequences more effectively than scrambled and unrelated sequences, with no significant differences compared to TRs. This suggests that compensatory strategies in developmental dyslexia operate at the sentence level rather than the individual word level. We found a tentative link between predive reading and SL in the SRT task. Not only was there a strong overlap between the neural networks that support prediction in reading and prediction in visual-motor learning, but also we found correlations between SL and reading ability and between SL and syntactic prediction ability. Finally, we found SL deficits for learning statistical patterns in the learning phase of the SRT task but not for using learned patterns in a subsequent recall task. More research is clearly needed but there seems to be a promising link between the ability to make linguistic predictions and SL in both normal and impaired reading.

## ACKNOWLEDGMENTS

The authors wish to thank Yufei Tan for her help for MRI acquisition for all the 50 subjects, and Leonardo Pinto Arata for his help with the R code for the LMM analysis. The authors also wish to thank all participants for their valuable contributions to this study. The work that led to this publication has been supported by AMPIRIC, a center of excellence on research in education and teacher training. Brain imaging was performed in the Center IRM-INT (UMR 7289, AMU-CNRS), platform member of France Life Imaging network (grant ANR-11-INBS-0006). The Centre de Calcul Intensif d’Aix-Marseille is gratefully acknowledged for providing access to its high-performance computing resources.

## FUNDING INFORMATION

Eddy Cavalli, Agence Nationale de la Recherche (https://dx.doi.org/10.13039/501100001665), Award ID: ANR-18-CE28-0006. Johannes C. Ziegler, Agence Nationale de la Recherche (https://dx.doi.org/10.13039/501100001665), Award ID: ANR-16-CONV-0002. Johannes C. Ziegler, Agence Nationale de la Recherche (https://dx.doi.org/10.13039/501100001665), Award ID: ANR-11-IDEX-0001-02. Jean-Luc Anton, Agence Nationale de la Recherche (https://dx.doi.org/10.13039/501100001665), Award ID: ANR-11-INBS-0006.

## AUTHOR CONTRIBUTIONS

**Elisa Gavard**: Conceptualization, Data curation, Formal analysis, Methodology, Writing – original draft, Writing – review & editing. **Valérie Chanoine**: Conceptualization, Formal analysis, Methodology, Writing – review & editing. **Franziska Geringswald**: Conceptualization, Formal analysis, Methodology, Writing – review & editing. **Jean-Luc Anton**: Conceptualization, Methodology, Writing – review & editing. **Eddy Cavalli**: Conceptualization, Funding acquisition, Writing – review & editing. **Johannes C. Ziegler**: Conceptualization, Funding acquisition, Methodology, Supervision, Writing – review & editing.

## DATA AND CODE AVAILABILITY

The data and the information needed to reproduce all the reported results and methodology are openly available at OSF (https://osf.io/twxj7/) and at OpenNeuro (https://openneuro.org/datasets/ds005341/versions/1.0.0; OpenNeuro Accession Number: ds005341).

## Supplementary Material


