# Supplementary material for: Neural Networks for Semantic and Syntactic Prediction and Visual-Motor Statistical Learning in Adult Readers With and Without Dyslexia
Source: Neurobiol Lang (Camb). 2025 Jul 22;6:nol.a.8. doi: 10.1162/nol.a.8 (PMC12328026; doi:10.1162/nol.a.8)
Supplement: Supplementary file 1 [file nol-6-1-8-s001.pdf]

Neural Networks for Semantic and Syntactic Prediction and Visuo-Motor Statistical Learning in Adult Readers with and without Dyslexia

Elisa Gavard, Valérie Chanoine, Franziska Geringswald, Jean-Luc Anton, Eddy Cavalli & Johannes C. Ziegler

SUPPLEMENTARY DATA

Univariate analysis of the fMRI data – whole brain results.

1. **Localizer task**

Supplementary Table S1. Localizer task – Contrast "Sentences reading - checkerboard" for all the participants. Regions were automatically labeled using Neuromorphometrics atlas in SPM12. Statistically significant effects on the whole-brain level were identified using a voxel-level threshold of p < .001 (uncorrected) and a cluster-level threshold of p < .05 (FWE-corrected for multiple comparisons). Stereotaxic coordinates for voxel (x, y, z) with maximal t-values within activation clusters are reported in the MNI standard space.

Brain Area	Extent	T values	Coordinates		
			x	y	z
L superior temporal gyrus	5415	11.20	-60	-36	4
R transverse temporal gyrus	3709	13.07	46	-10	16
L precentral gyrus	799	10.48	-56	-8	50
R precentral gyrus	67	4.96	4	-26	50
L supplementary motor cortex	3734	10.83	-2	-0	68
L precuneus	224	8.04	-30	-54	8
R precuneus	308	8.69	28	-48	12
	109	4.98	10	-66	38
L caudate	541	6.04	-8	-0	22
L cerebellum exterior	172	5.43	-24	-68	-58
	44	4.54	-44	-60	-28
R cerebellum exterior	1336	10.25	30	-68	-56
	136	8.28	32	-62	-24

Note. L: left hemisphere, R: right hemisphere.

2. **Predictive reading task**

Supplementary Table S2. Predictive reading task – Contrast "Semantic prediction (related-scrambled)" for all the participants. Regions were automatically labeled using Neuromorphometrics atlas in SPM12. Statistically significant effects on the whole-brain level were identified using a voxel-level threshold of $p < .001$ (uncorrected) and a cluster-level threshold of $p < .05$ (FWE-corrected for multiple comparisons). Stereotaxic coordinates for voxel (x, y, z) with maximal t-values within activation clusters are reported in the MNI standard space.

Brain Area	Extent	T values	Coordinates		
			x	y	z
L middle frontal gyrus	676	5.97	-44	24	28
	63	4.66	-46	8	54
L IFG (p. triangularis)	472	5.40	-40	42	2
L middle temporal gyrus	233	5.20	-58	-48	-10
L inferior temporal gyrus	63	5.93	-44	-60	-8
R inferior temporal gyrus	82	4.81	44	-56	-12
L supramarginal gyrus	73	4.85	-48	-46	48
L angular gyrus	337	5.46	-34	-64	46
R angular gyrus	132	4.76	34	-64	48
L inferior occipital gyrus	137	4.89	-26	-94	-10
L caudate	45	4.09	-12	6	6
R occipital pole	117	5.61	26	-98	-8

Note. L: left hemisphere, R: right hemisphere.

Supplementary Table S3. Predictive reading task – Contrast "Syntactic prediction (related-scrambled)" for all the participants. Regions were automatically labeled using Neuromorphometrics atlas in SPM12. Statistically significant effects on the whole-brain level were identified using a voxel-level threshold of $p < .001$ (uncorrected) and a cluster-level threshold of $p < .05$ (FWE-corrected for multiple comparisons). Stereotaxic coordinates for voxel (x, y, z) with maximal t-values within activation clusters are reported in the MNI standard space.

Brain Area	Extent	T values	Coordinates		
			x	y	z
L superior frontal gyrus	46	4.69	-12	56	38
	47	4.22	-4	40	46
L IFG (p. triangular)	950	5.73	-46	34	-2
L IFG (p. orbital)	950	5.63	-50	18	-14
R frontal operculum	280	5.38	48	22	-4
L temporal pole	950	7.73	-52	14	-24
	59	4.76	-36	4	-36
L inferior temporal gyrus	36	4.80	-44	-14	-28
R superior temporal gyrus	280	4.83	52	-0	-18
R middle temporal gyrus	75	4.48	50	-20	-12
R cuneus	51	4.30	6	-84	16
R lingual gyrus	47	4.94	12	-76	-4
L posterior insula	41	5.14	-30	-24	4
L putamen	51	5.38	-20	4	6

Note. L: left hemisphere, R: right hemisphere.

Supplementary Table S4. Predictive reading task – Contrast "Semantic – Syntactic prediction (related-scrambled)" for all the participants. Regions were automatically labeled using Neuromorphometrics atlas in SPM12. Statistically significant effects on the whole-brain level were identified using a voxel-level threshold of $p < .001$ (uncorrected) and a cluster-level threshold of $p < .05$ (FWE-corrected for multiple comparisons). Stereotaxic coordinates for voxel (x, y, z) with maximal t-values within activation clusters are reported in the MNI standard space.

Brain Area	Extent	T values	Coordinates		
			x	y	z
L superior frontal gyrus	225	5.40	-2	30	48
R posterior frontal gyrus	360	5.60	30	-2	66
L middle frontal gyrus	471	6.09	-50	12	50
	73	5.49	-44	58	2
L frontal operculum	148	5.39	-38	22	10

L central operculum	68	4.57	-46	2	12
R frontal operculum	194	6.55	42	22	10
R precentral gyrus	89	5.04	54	4	16
	49	4.53	2	-24	58
	55	4.43	46	-12	56
L postcentral gyrus	182	5.59	-18	-28	76
	60	5.44	-54	-14	56
R postcentral gyrus	183	5.75	16	-30	72
	121	4.88	24	-40	74
	62	4.59	52	-30	58
L supplementary motor cortex	185	5.11	-6	6	56
R anterior insula	80	6.30	46	10	-10
	69	4.62	36	8	14
L superior parietal lobule	151	4.90	-36	-40	66
R superior parietal lobule	49	4.74	18	-58	70
	129	4.55	42	-50	60
L angular gyrus	94	4.32	-64	-54	34
L temporal pole	63	4.99	-54	8	-24
	62	4.66	-46	10	-12
L middle temporal gyrus	67	4.54	-60	-40	-4
R middle temporal gyrus	93	4.87	62	-4	-30
L precuneus	77	4.69	-26	-50	8
L posterior cingulate gyrus	88	4.94	-2	-46	-0
R anterior cingulate gyrus	83	4.36	4	40	20
R caudate	1842	7.53	4	10	14
L cerebellum exterior	76	5.06	-44	-60	-50
R cerebellum exterior	128	4.78	2	-68	-14

Note. L: left hemisphere, R: right hemisphere.

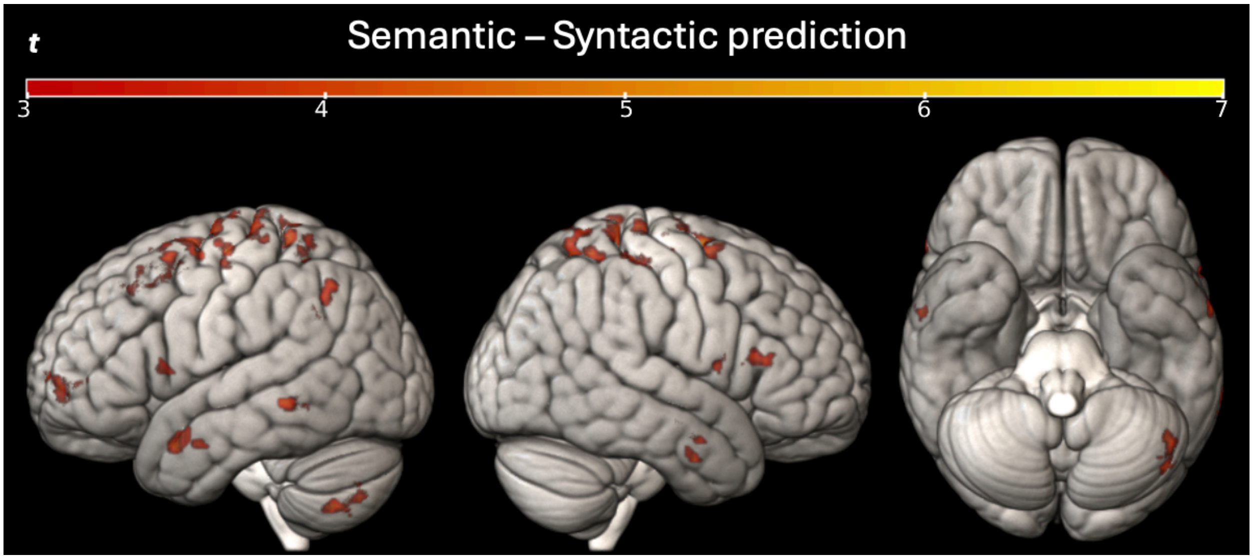

Supplementary Figure S1. Univariate whole-brain results of reading in Predictive reading task (semantic – syntactic prediction) for all the participants. Statistical t-maps for dyslexic readers (DRs, n = 24) and typical readers (TRs, n = 25) are projected respectively on left, right, and below side of cortical surfaces (from MNI standard human cortex) using an uncorrected voxel-wise threshold of $p < 0.001$ and a cluster-wise threshold with FWE correction of $p < 0.05$.

Supplementary Table S5. Predictive reading task – Contrast "Syntactic – Semantic prediction (related-scrambled)" for all the participants. Regions were automatically labeled using Neuromorphometrics atlas in SPM12. Statistically significant effects on the whole-brain level were identified using a voxel-level threshold of $p < .001$ (uncorrected) and a cluster-level threshold of $p < .05$ (FWE-corrected for multiple comparisons). Stereotaxic coordinates for voxel (x, y, z) with maximal t-values within activation clusters are reported in the MNI standard space.

Brain Area	Extent	T values	Coordinates		
			x	y	z
R precuneus	110	4.82	4	-52	48
L lingual gyrus	61	4.67	-8	-82	-8
R lingual gyrus	782	5.14	8	-76	8
	46	4.99	26	-58	4

Note. L: left hemisphere, R: right hemisphere.

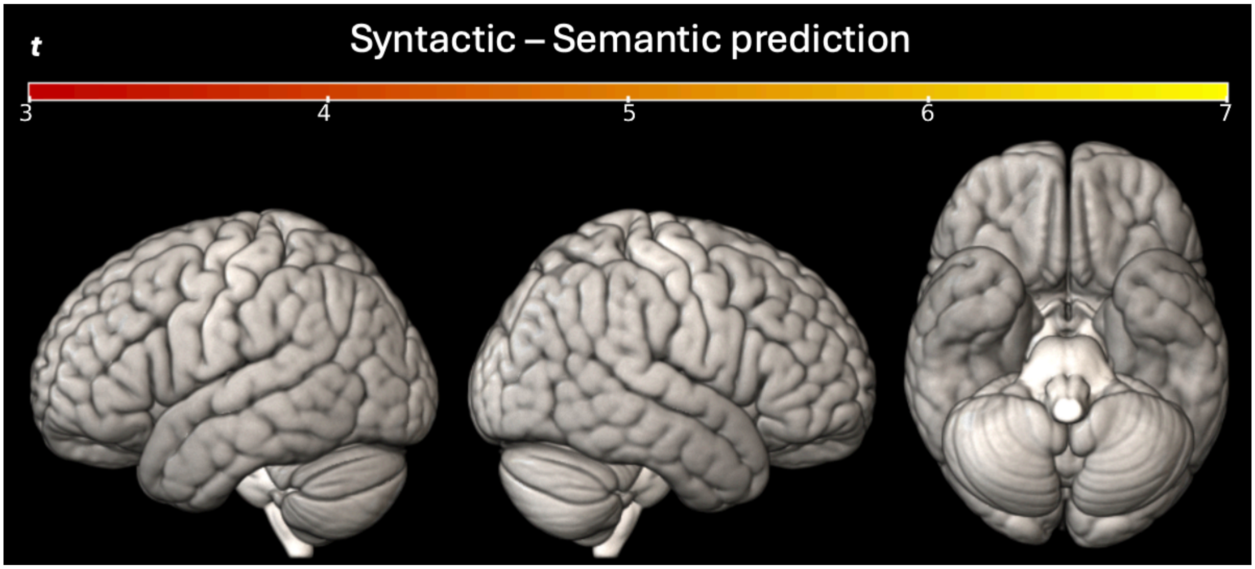

Supplementary Figure S2. Univariate whole-brain results of reading in Predictive reading task (syntactic – semantic prediction) for all the participants. Statistical t-maps for dyslexic readers (DRs, n = 24) and typical readers (TRs, n = 25) are projected respectively on left, right, and below side of cortical surfaces (from MNI standard human cortex) using an uncorrected voxel-wise threshold of $p < 0.001$ and a cluster-wise threshold with FWE correction of $p < 0.05$.

3. Serial Reaction Time task

Supplementary Table S6. Serial Reaction Time task – Contrast "Learned – Randomized sequences" for all the participants. Regions were automatically labeled using Neuromorphometrics atlas in SPM12. Statistically significant effects on the whole-brain level were identified using a voxel-level threshold of $p < .001$ (uncorrected) and a cluster-level threshold of $p < .05$ (FWE-corrected for multiple comparisons). Stereotaxic coordinates for voxel (x, y, z) with maximal t-values within activation clusters are reported in the MNI standard space.

Brain Area	Extent	T values	Coordinates		
			x	y	z
L superior frontal gyrus	1788	6.33	-18	60	10
	461	5.78	-24	30	58
	347	4.97	-10	42	18
L middle frontal gyrus	100	5.15	-44	12	56
L inferior frontal gyrus	157	4.21	-56	-12	-20

L precentral gyrus	850	6.40	-4	-32	48
L postcentral gyrus	100	5.33	-22	-28	70
R postcentral gyrus	106	5.04	22	-30	64
R lingual gyrus	55	5.31	12	-38	-4
L angular gyrus	1336	6.95	-56	-66	42
L middle temporal gyrus	556	5.30	-64	-36	-10
	157	5.24	-58	-4	-28
R middle temporal gyrus	67	4.13	66	-4	-14
R cuneus	288	5.95	4	-80	40
L thalamus	233	6.11	-16	-36	4
R thalamus	180	6.09	16	-32	22
L amygdala	2212	6.59	-18	-2	-20
L caudate	162	6.21	-18	-30	26
R caudate	138	5.99	18	18	14
R putamen	602	6.28	18	10	-8
L cerebellum exterior	53	4.59	-20	-88	-38
	57	4.40	-46	-74	-36
R cerebellum exterior	1365	6.12	40	-78	-38

Note. L: left hemisphere, R: right hemisphere.
